# Pseudouridine-Modifying Enzymes SapB and SapH Control Entry into the Pseudouridimycin Biosynthetic Pathway

**DOI:** 10.1021/acschembio.2c00826

**Published:** 2023-04-03

**Authors:** Erika Artukka, Robert Schnell, Kaisa Palmu, Petja Rosenqvist, Edit Szodorai, Jarmo Niemi, Pasi Virta, Gunter Schneider, Mikko Metsä-Ketelä

**Affiliations:** † Department of Life Technologies, University of Turku, FIN-20014 Turku, Finland; ‡ Department of Chemistry, University of Turku, FIN-20014 Turku, Finland; § Department of Neuroscience, Karolinska Institutet, SE-17177 Stockholm, Sweden; ∥ Department of Medical Biochemistry and Biophysics, Karolinska Institutet, SE-17177 Stockholm, Sweden

## Abstract

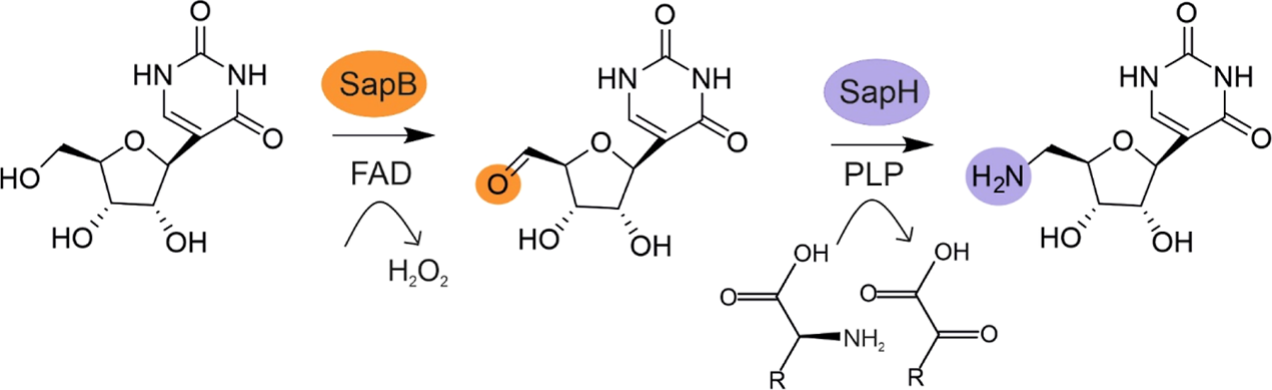

Pseudouridimycin is a microbial *C*-nucleoside natural product that specifically inhibits bacterial RNA polymerases by binding to the active site and competing with uridine triphosphate for the nucleoside triphosphate (NTP) addition site. Pseudouridimycin consists of 5′-aminopseudouridine and formamidinylated, N-hydroxylated Gly–Gln dipeptide moieties to allow Watson–Crick base pairing and to mimic protein–ligand interactions of the triphosphates of NTP, respectively. The metabolic pathway of pseudouridimycin has been studied in *Streptomyces* species, but no biosynthetic steps have been characterized biochemically. Here, we show that the flavin-dependent oxidase SapB functions as a gate-keeper enzyme selecting pseudouridine (*K*_M_ = 34 μM) over uridine (*K*_M_ = 901 μM) in the formation of pseudouridine aldehyde. The pyridoxal phosphate (PLP)-dependent SapH catalyzes transamination, resulting in 5′-aminopseudouridine with a preference for arginine, methionine, or phenylalanine as cosubstrates as amino group donors. The binary structure of SapH in complex with pyridoxamine-5′-phosphate and site-directed mutagenesis identified Lys289 and Trp32 as key residues for catalysis and substrate binding, respectively. The related *C*-nucleoside oxazinomycin was accepted as a substrate by SapB with moderate affinity (*K*_M_ = 181 μM) and was further converted by SapH, which opens possibilities for metabolic engineering to generate hybrid *C*-nucleoside pseudouridimycin analogues in *Streptomyces*.

Natural products derived from the secondary metabolism of bacteria are an important source of antibiotics, anticancer agents, and other drugs.^1^ Approximately two-thirds of antibiotics are natural products or their semi-synthetic derivatives.^2^ Soil-dwelling actinomycetes have been a rich source of antibiotics and have provided, among others, streptomycin, tetracycline, erythromycin, and rifamycin that are in clinical use.^3^ However, the emergence and spread of bacterial pathogens resistant to all antibiotics is of great concern, particularly as recent years have seen a decline in the antibiotics discovery pipeline and introduction of new molecules into clinical practice.^4^

RNA polymerase (RNAP) is an essential enzyme in all kingdoms of life and an established target for antibacterial therapy.^5^ The therapeutic window is provided by significant differences between bacterial RNAPs and the three eukaryotic, human, RNAP enzymes.^6^ In addition, bacterial RNAP proteins are evolutionary conserved and therefore provide opportunities for broad-spectrum activities.^6^ Two classes of RNAP inhibitors are currently in clinical use. Rifamycin and its semi-synthetic derivatives, which are particularly effective against mycobacteria, prevent extension of short RNA products by binding adjacent to the active site.^7,8^ Fidaxomicin is a narrow spectrum antibiotic against *Clostridium difficile* that allosterically inhibits RNAP–DNA interactions.^9^

Nucleoside analogues are widely used as antiviral, antimicrobial, and anticancer agents.^10^ Most compounds in clinical use are *N*-nucleosides, which may be susceptible to loss-of-activity through cleavage of the *N*-glycosidic bond.^11^ This has raised considerable interest in hydrolysis-resistant *C*-nucleosides,^11^ such as the microbial natural products pseudouridimycin (**1**),^12,13^ oxazinomycin (**2**),^14^ showdomycin (**3**),^15^ pyrazofurin (**4**),^16,17^ and formycin (**5**) (Figure 1).^16,17^ Particularly, **1** is a selective inhibitor of bacterial RNAP with a novel mode-of-action.^12^ The structure of **1** can be considered to be composed of two units, a 5′-aminopseudouridine nucleoside that forms Watson–Crick base pairing with adenine and a formamidinylated, N-hydroxylated Gly–Gln dipeptide moiety that mimics the interactions that naturally occur between the triphosphates of nucleotides and RNAP.^12^ These characteristics allow competitive binding of **1** in place of uridine triphosphate and potent inhibition of transcription of bacterial multi-subunit RNAPs.^12^ The triphosphate of **2** has also been shown to bind to the active site of RNAP and arrests transcription at polythymidine sequences *in vitro* but unlike **1** does not differentiate between bacterial and eukaryotic RNAPs.^18^

**Figure 1:**
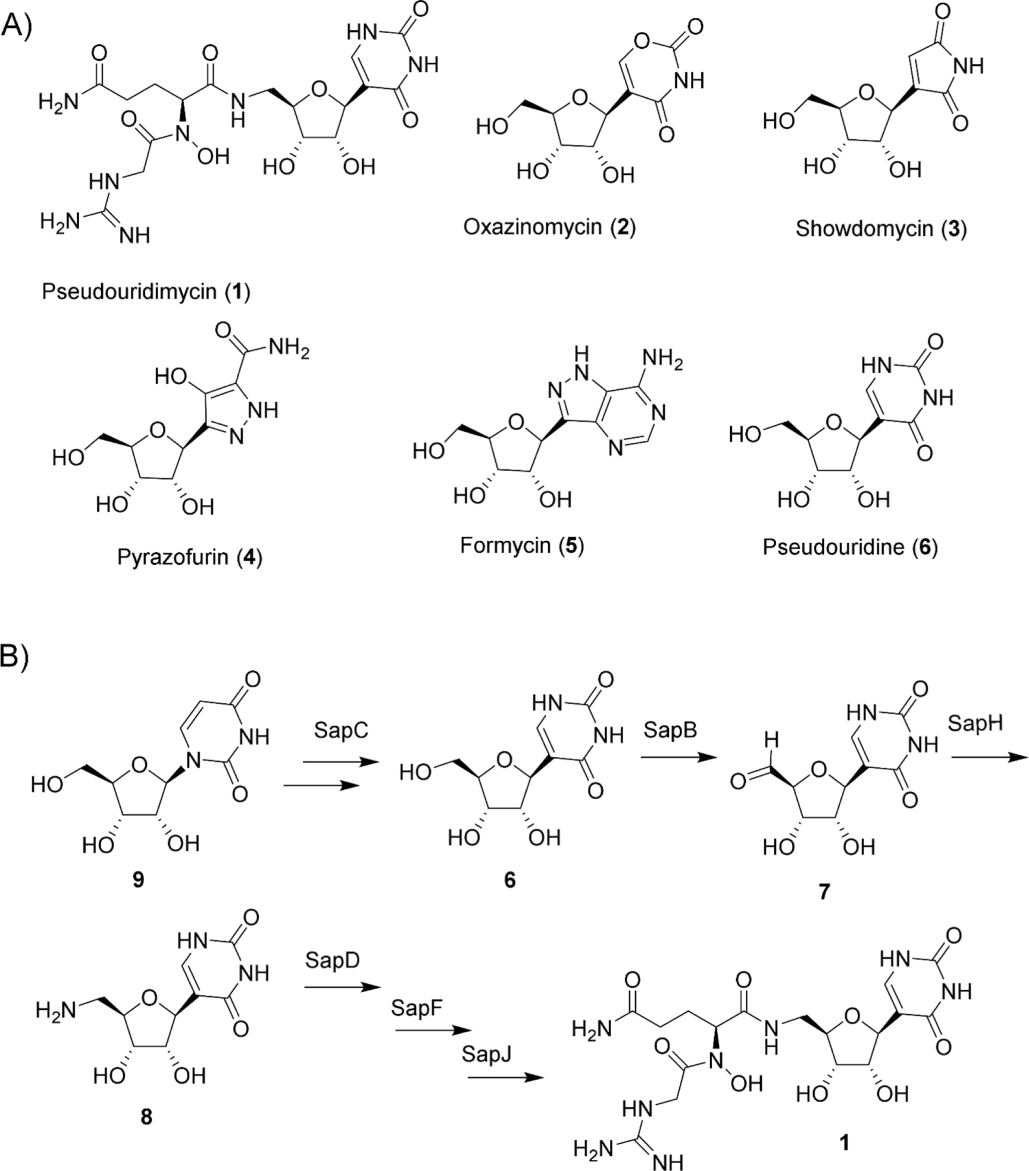
(A) Examples of natural product *C*-nucleosides. (B) Pseudouridimycin biosynthesis pathway.

The biosynthetic gene cluster (BGC) of **1** has been identified as being produced by *Streptomyces* sp. ID38640 (*pum* BGC)^13^ and *Streptomyces albus* DSM 40763 (*sap* BGC).^19^ Gene inactivation and heterologous expression studies in *Streptomyces* sp. ID38640 have uncovered eight genes that code for enzymes responsible for the formation of **1**.^13,20,21^ The pseudouridine synthase *pumJ*(13) and adenylate kinase *pumH*(20,21) have been proposed to provide pseudouridine (**6**) or a phosphorylated derivative of **6** for the pathway. Conversion to pseudouridine aldehyde (**7**) *via* alcohol oxidation by PumI (SapB in *S. albus* DSM 40763) and subsequent amine formation to 5′-aminopseudouridine (**8**) by PumG (SapH in *S. albus* DSM 40763) are the likely sequential biosynthetic steps.^13,19^ The ATP-grasp ligase PumK has been proposed to attach glutamine to **8**, which is followed by N-hydroxylation by the flavoenzyme PumE.^13,19^ The final steps include biosynthesis and attachment of guanidinoacetate by PumN and PumM, respectively.^13,19^

Here, we present the biochemical characterization of the early biosynthetic pathway of **6**. We show that **6** is a true intermediate on the pathway and that the flavoenzyme SapB functions as a gatekeeper enzyme to generate **7** while preventing the formation of uridine congeners of **6**. We have solved the crystal structure of SapH and demonstrate that the protein completes the formation of **8**. Detailed understanding of the biosynthesis of **6** will facilitate metabolic engineering efforts for the generation of biosynthetic derivatives of this promising antibiotic.

## Results

### Production and Purification of SapB and SapH

Bioinformatic analysis of the *sap* BGC indicated that SapB, a flavoenzyme of the glucose–methanol–choline oxidoreductase family, and SapH, a PLP-dependent transaminase, could catalyze early steps in pseudouridimycin biosynthesis. The proteins were produced as N-terminally histidine-tagged enzymes in *Escherichia coli* TOP10 from synthetic codon-optimized genes. Single-step purification to near homogeneity was achieved by affinity chromatography. Ultraviolet–visible spectroscopy (UV/vis) spectra of SapB and SapH were typical for flavin and PLP-containing enzymes (Figure S1A), respectively. Furthermore, liquid chromatography–mass spectrometry (LC/MS) analysis identified the SapB cofactor to be flavin adenine dinucleotide (FAD) (Figure S1B).

### Enzymatic Activities of SapB and SapH

**Figure 2:**
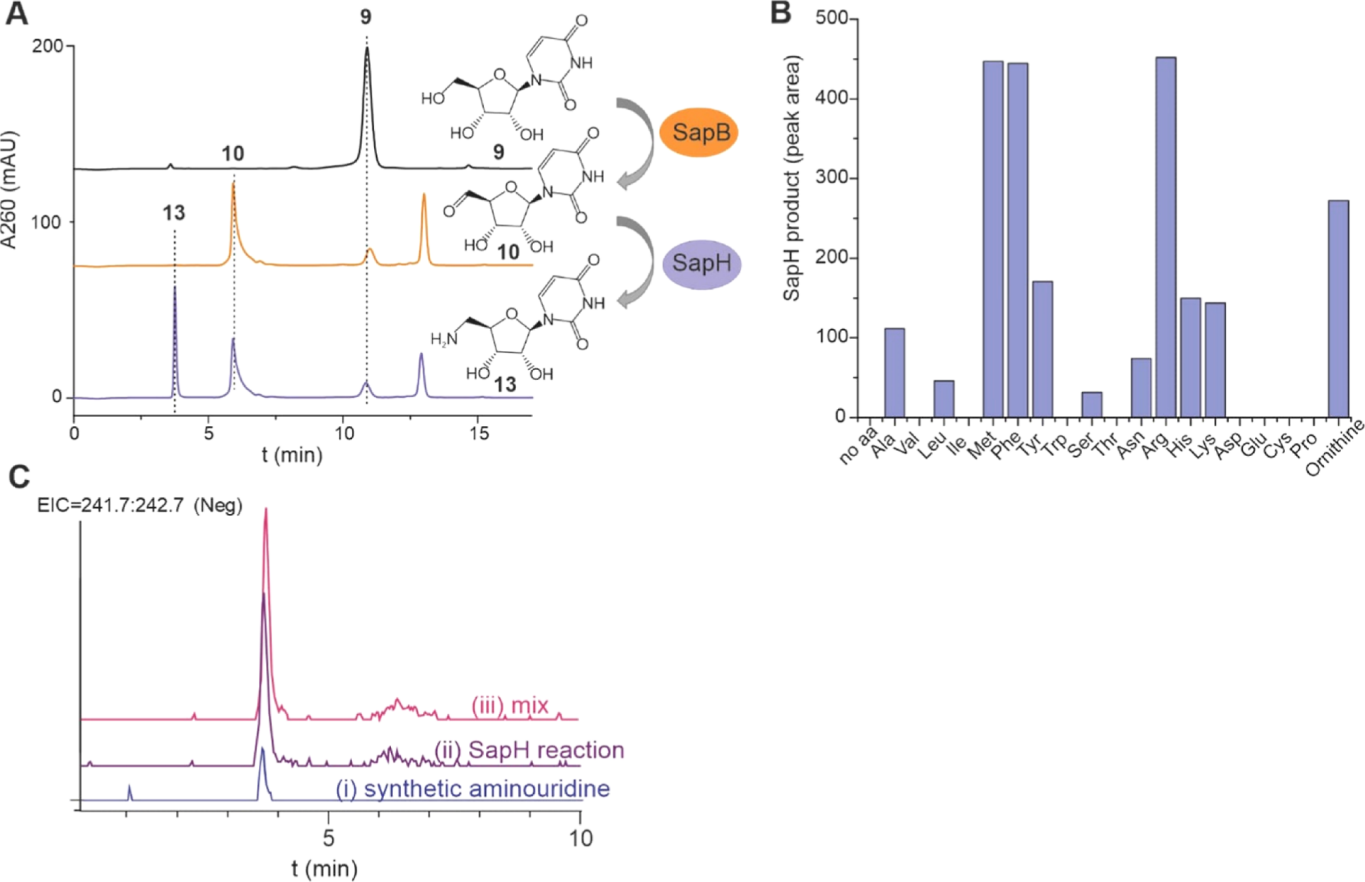
Reactions with SapB and SapH with uridine as a substrate. (A) HPLC data of SapB and SapH reactions with uridine. SapB reaction included 1.5 mM uridine and 1 μM SapB. Reaction with SapH included 1.5 mM uridine, 1 μM SapB, 2 μM SapH, and 5 mM Arg. (B) Relative SapH activity with different amino acids was analyzed by HPLC. Reactions included 1.5 mM uridine, 1 μM SapB, 2 μM SapH, and 5 mM amino acid. Ornithine was measured separately, and reaction with Arg was used to compare the activity. (C) LC/MS data with (i) 400 μM synthetic aminouridine, (ii) SapH reaction with 2 μM SapH, 1.5 mM uridine, 5 mM Arg, and 1 μM SapB, and (iii) SapH reaction + 200 μM aminouridine.

For initial activity assays, we utilized uridine (**9**) as a substrate for SapB and SapH and analyzed reaction products with high-performance liquid chromatography (HPLC) (Figure 2A), LC/MS (Figure S2), and high-resolution electrospray ionization–mass spectrometry (HRESI-MS) (Figures S3–S7). The SapB reaction led to the formation of several new product peaks, where the main product corresponded to 5′-aldehyde uridine (**10**) with a −2 Da decrease in mass to the substrate **9**. Consistently, the aldehyde product **10** was found in equilibrium with a diol form (**11**) corresponding to masses of 242 and 260 (Figure S2), respectively. Finally, a minor product **12** possibly conforming to a congener with a carboxyl group in the 5′-position with a mass of 258 was detected (Figure S2). Compound **12** could conceivably be formed from **11***via* further oxidation of the diol by SapB. In agreement with other members of the glucose–methanol–choline oxidoreductase family, SapB did not require any cosubstrates for alcohol oxidation, but the reaction resulted in the coproduction of H_2_O_2_ (see below). The result confirmed that **11** is oxidized during the reductive half-reaction of the flavin cycle, and the resting state of SapB is restored *via* reaction of flavin with molecular oxygen and subsequent release of H_2_O_2_.

Addition of the transaminase SapH and amino acid donor cosubstrates to the SapB reaction led to the formation of another new product **13** (Figure 2A), which corresponded to 5′-amino-5′-deoxyuridine with a mass of 243.09 (Figure 2). Next, we tested all 20 proteinogenic amino acids and ornithine as cosubstrates at 5 mM concentration for the transamination reaction. SapH accepts 11 amino acids as well as ornithine as cosubstrates but shows preference for methionine, phenylalanine, and arginine (Figure 2B). The two reactions could also be decoupled and conversion of **10** to **13** was detected when the supernatant from a SapB reaction was filtered (Amicon Ultra, 3 kDa molecular weight cut-off) to remove proteins and used as a substrate for an individual SapH reaction (Figure S8A).

In order to confirm the identity of the SapB and SapH reaction product, we synthesized **13** (Supporting Information) from **9** in three steps *via* initial tosylation of the unprotected nucleoside. The 5′-*O*-tosyl uridine was then treated with sodium azide, affording 5′-azido-5′-deoxyuridine. The azidouridine was finally converted to **13** by Staudinger reduction. The synthetic **13** (Figures S9–S11) was found to have the same retention time as the enzymatic reaction product **13** and co-elute as a single peak in LC/MS (Figure 2C). The experiment confirmed that SapB and SapH use nucleosides as substrates and convert them to their 5′-aldehyde and 5′-amino congeners, respectively.

**Figure 3:**
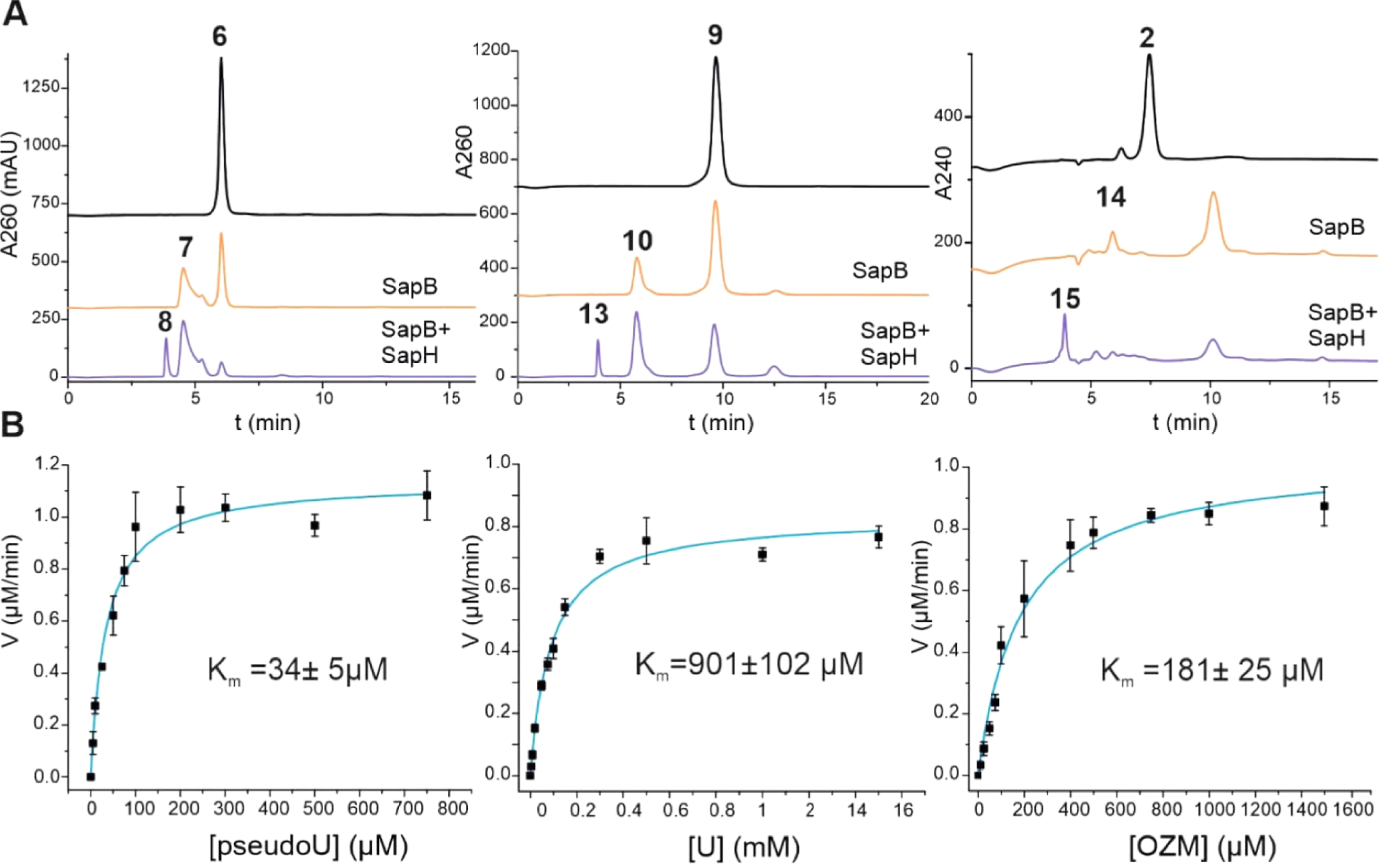
Substrate promiscuity of SapB and SapH and kinetic analysis of the reaction catalyzed by SapB. (A) SapB and SapB + SapH reactions with different substrates **6**, **9**, and **2** were analyzed with HPLC. Reactions included 1.5 mM substrate, 5 mM arginine, and 1 μM enzymes and were incubated for 20 h. (B) SapB kinetics with different substrates were measured by following the reaction of H_2_O_2_ with HRP and ABTS.

Next, we carried out reactions with the natural substrate **6** that led to the formation of products **7** and **8** based on HPLC-UV/vis (Figure 3) and HR-MS (Figures S12–S14). Decoupling of the reactions showed the conversion of **6** to **7** (Figure S8B). Similarly, the *C*-nucleoside antibiotic **2** was converted to products **14** and **15** (Figures 3 and S15–S17). The substrate scope of SapB appeared to be relatively strict since no enzymatic activity was detected with the *C*-nucleosides **3–5**.

We performed kinetic analysis of SapB based on the measurement of H_2_O_2_ formation using horseradish peroxidase (HRP) and oxidation of 2,2′-azino-bis(3-ethylbenzthiazoline-6-sulfonic acid) (ABTS) at *A*_405nm_ (Table 1). *K*_M_ values determined from Michaelis–Menten plots (Figure 3B) revealed that SapB harbored a 30-fold higher affinity toward **6** (*K*_M_ = 34 μM) than **9** (*K*_M_ = 901 μM). This implies that SapB functions as a gatekeeper enzyme to prevent the formation of congeners derived from the naturally abundant **9**. Encouragingly, the moderate affinity toward **2** (*K*_M_ = 181 μM) indicates that it may be possible to use metabolic engineering to generate hybrid pseudouridimycin derivatives with alternate base units. The modest catalytic rate of SapB reaction (1 min^–1^) might be the result of slow FAD cofactor regeneration or if FAD was present only in a fraction of recombinantly produced SapB. However, addition of free FAD to the reaction mixture did not affect the reaction rate, indicating that FAD is tightly bound into the enzyme. Although the SapB reaction is slow, all of the substrate was converted to the product during an overnight reaction.

**Table 1:** Basic Kinetic Parameters for SapB with **2**, **6**, and **9** as Substrates

substrate	*K*_M_ (μM)	*k*_cat_ (min^–1^)	*k*_cat_/*K*_M_ (μmol^–1^ min^–1^)
pseudouridine (**6**)	34 ± 5	1.14	3.3 × 10^–2^
oxazinomycine (**2**)	181 ± 25	1.03	5.7 × 10^–3^
uridine (**9**)	901 ± 102	0.833	9.2 × 10^–4^

### Three-Dimensional Structure of SapH

**Figure 4:**
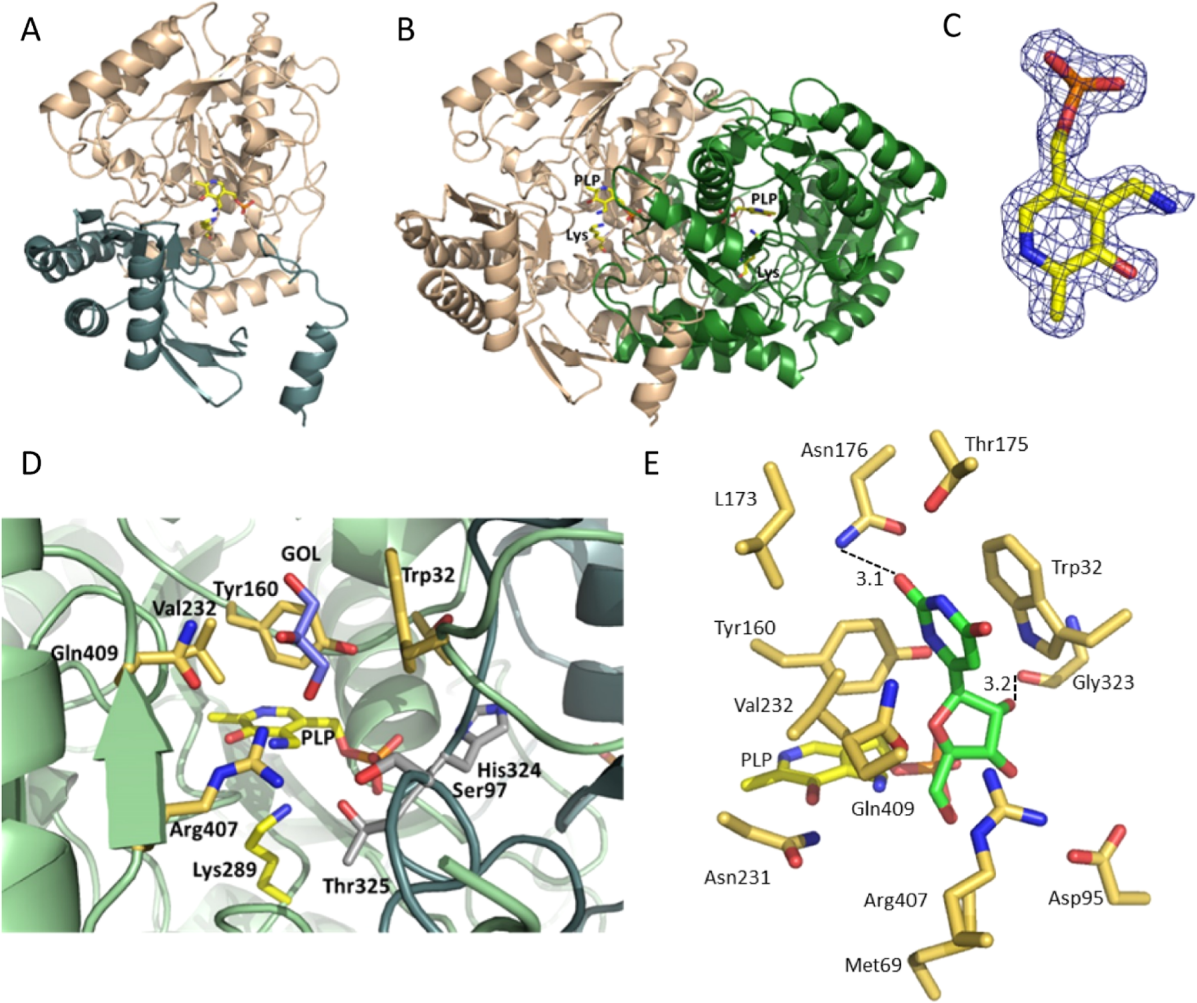
Crystal structure of SapH and architecture of the active-site cavity. (A) SapH subunit is shown in cartoon representation colored according to the domain structure. The pyridoxalamine form of the PLP cofactor and the invariant Lys289 are depicted as sticks. (B) SapH dimer in cartoon representation, showing the subunits in beige and green colors. PLP and the conserved Lys289 depicted as sticks. (C) 2*F*_o_ – *F*_c_ electron density map, contoured at 1.0 σ for the bound pyridoxalamine. (D) Active-site cavity of SapH with residues forming the substrate binding cavity depicted as sticks. The pyridoxalamine form of the PLP cofactor and the invariant Lys289 are colored yellow, the glycerol molecule located at this cavity is in purple, amino acid side chains from one of the subunits are shown in beige, and residues provided by the second subunit of the dimer are shown in gray. (E) Stereo view image of the putative binding mode of **7** in the active site of SapH based on manual docking.

The structure of SapH was determined to a 1.55 Å resolution by X-ray crystallography (Table S1) in space group *P*2_1_2_1_2_1_ using molecular replacement. The asymmetric unit of the crystals contains a tightly packed dimer, and each polypeptide chain shows the fold characteristic for PLP enzymes of the fold type I, subclass II.^22^ The subunit consists of two domains, each of the α/β type. Residues 1–74 and 347–440 form a smaller domain, containing an anti-parallel three-stranded and anti-parallel four-stranded β-sheet, the latter flanked by α-helices on one side, whereas the dominating feature of the larger domain (residues 75–346) is a mixed seven-stranded β-sheet, flanked on both sides by α-helices (Figure 4A).

The overall fold and the mode of dimerization seen in SapH is essentially identical to what is observed in the fold-type I aminotransferase family, for instance, in the related 7,8 diamino pelargonic acid synthase^23^ and 8-amino-7-oxononaoate aminotransferase^24^ (Figure 4B). The two individual chains of the dimer, related by two-fold non-crystallographic symmetry, are structurally very similar as indicated by the root-mean-square deviation of the polypeptide Cα atoms of 0.2 Å over 421 aligned residues. The buried surface area in the dimerization interface accounts for 4910 Å^2^, corresponding to a 26% surface area in each monomer. The dimer observed in the SapH crystals most likely represents the biologically active form of the enzyme as the cofactor binding site, and the active-site pocket is built up by residues from both chains constituting the dimer (Figure S18).

The binding site of the PLP cofactor is located close to the dimer interface, with residues from both subunits involved in cofactor–protein interactions (Figure 4D). The bound PLP was modeled as pyridoxamine, according to the observed electron density map (Figure 4C), and because the expected covalent link between PLP and Lys289, conserved in the protein family, is absent in the crystal structure (Figure 4C,D). The phosphate group of PLP is held in place by hydrogen bonds to the side chain of Ser128, the main-chain nitrogen atom of Gly128 and the side chains of His324 and Thr325. The latter two residues are located in a loop region of the second subunit that forms part of the PLP binding site and the active-site cavity. The aromatic ring system of PLP is sandwiched between the hydrophobic side chains of Tyr160 and Val262. The phenolic oxygen atom of PLP forms a hydrogen bond to the side chain of Asn23, and the ring nitrogen is hydrogen-bonded to the side chain of Asp260. The latter interaction is crucial for catalysis by PLP-dependent enzymes (Figure S19).

Extending from the bound pyridoxamine 5′-phosphate (PMP) to the bulk solution, a pocket is formed that represents the active-site cavity formed by residues from both subunits and lined with hydrophobic and polar residues that might be involved in substrate binding and catalysis (Figure 4D). At the opening of this cleft is a flexible disordered loop region (residues 35–43) that could act as a flap and close the active site once the substrate is bound. The electron density map indicated the presence of a compound bound in this cavity which was modeled as a glycerol molecule that was a component in the storage buffer of the purified protein used for crystallization.

### Binding of the Pseudouridine Aldehyde Substrate **7** in the SapH Active Site

The crystal structure of SapH allowed manual docking of substrate **7** into the active site at the position of the glycerol molecule (Figures 4E and S19). In the proposed binding mode, the uracyl ring is stacking against Trp32 with interplane distances of 3.9–4.0 Å, the 6′-oxo-group of the uracyl ring makes a potential hydrogen bond with amide nitrogen of Asn176, and the 2′-OH of ribose forms another potential hydrogen bond with the carbonyl group of Gly323. The docking pose places the 5′-carbonyl group of the substrate perpendicular to the plane of the cofactor pyridine ring in a position favorable for catalysis. Next, we performed site-directed mutagenesis to verify the putative binding mode of the substrate. Amino acid residues Lys289 and Trp32 were chosen to confirm their essential roles for catalysis and substrate binding, respectively. Consistent with this model, SapH W32A and SapH K289H variant enzymes were both inactive and changes in the UV/vis spectrum indicated that the variants may not able to correctly bind the cofactor PLP or that binding might be severely impeded (Figure S20).

## Discussion

Pseudouridimycin (**1**) is a promising new antibiotic that inhibits bacterial transcription by binding to the active site of bacterial RNAP.^12^ Structurally, **1** belongs to the class of *C*-nucleoside antibiotics but has a unique formamidinylated, N-hydroxylated Gly–Gln dipeptide appended to the 5′-position of the ribose unit. These two features are the basis for the novel mode of action of **1**. The ability to bind directly to the active site of RNAP can be considered to be of great importance since this delays the development of antibiotic resistance. The rate of spontaneous development of resistance to **1** was found to be an order of magnitude lower than that of clinically used rifampin.^12^ In this work, we provide first biochemical and structural evidence for pseudouridimycin biosynthesis and demonstrate that SapB and SapH catalyze pseudouridine (**6**)-modifying reactions. We show *in vitro* that SapB and SapH catalyze sequential reactions that lead to the formation of pseudouridine aldehyde (**7**) and 5′-aminopseudouridine (**8**), respectively.

**Figure 5:**
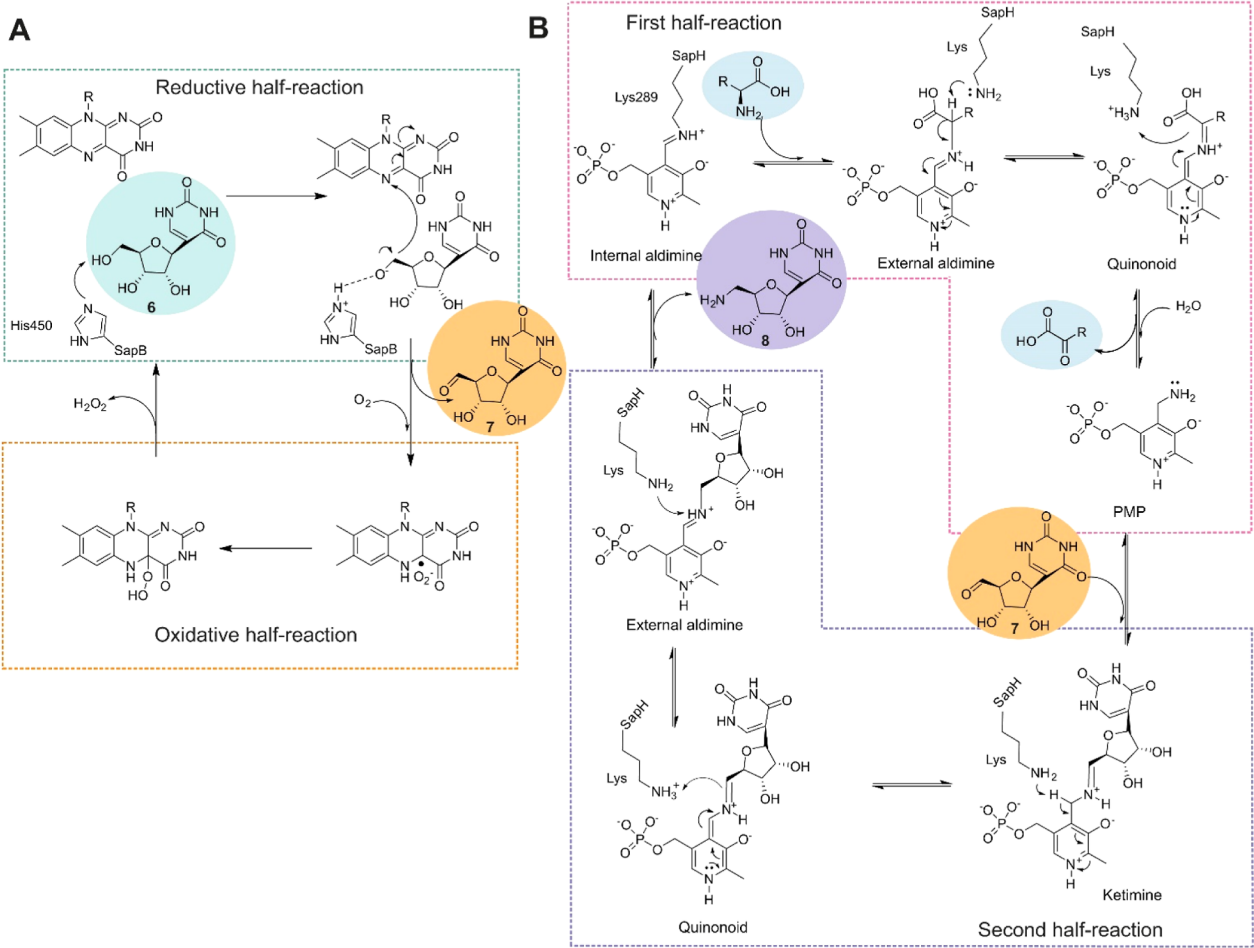
Proposed reaction mechanisms for (A) SapB and (B) SapH.

We propose that the SapB reaction follows a catalytic cycle similar to that of other enzymes in the glucose–methanol–choline enzyme family (Figure 5A). Catalysis is based on classical flavin chemistry that consists of reductive and oxidative half reactions.^25^ During the reductive half reaction, an active-site histidine acts as a catalytic base^26^ to abstract the proton from the 5′-OH of the substrate **6**. Two electrons are subsequently transferred from **6** to form reduced FAD and **7**. Then the reduced FAD reacts with molecular oxygen to form oxidized FAD and H_2_O_2_.^27^ Based on sequence alignment (Figure S21), we propose that conserved His450 residue of SapB may have an important role in the catalysis, but challenges in structure determination of the enzyme have prevented verification of the hypothesis.

Our combined biochemical and structural data suggest that the transamination reaction catalyzed by SapH most likely follows a reaction mechanism typical for PLP-dependent transaminases (Figure 5B). We propose that PLP is bound to the SapH enzyme *via* Lys289, forming a Schiff base and an internal aldimine. Then, during the first half-reaction, an external aldimine is formed upon the binding of an amino donor such as arginine. The amino group is transferred to PLP, forming the aminated cofactor PMP *via* a quinonoid intermediate. In the second half-reaction, the ketoacid is replaced by substrate **7**, and the amino group is transferred *via* ketimine and quinonoid intermediates. In the final catalytic step, the external aldimine is again converted to an internal aldimine by Lys289, and the formed product **8** is released. The ability of SapH to catalyze the conversion of non-cognate **10** to **13** is noteworthy, since this is the natural reaction of the aminotransferase LipO (37.7% sequence identity) on the biosynthetic pathways of several peptidyl nucleoside antibiotics.^28^

One open question in the biosynthesis of **1** has been the origin of the nucleoside substrate and the phosphorylation state of early pathway intermediates. Pseudouridine synthases such as PumJ typically use tRNAs as substrates and generate phosphorylated products. Pseudouridimycin BGC also encodes an adenylate kinase homologue PumH of unknown function. Similarly, the biosynthesis of the nucleosides nikkomycin, polyoxin, and malayamycin revealed cryptic phosphorylation and dephosphorylation steps.^29,30^ These factors have led to proposals that pseudouridine 2′-phosphates may be the substrates for the 5′-transamination step in pseudouridimycin biosynthesis.^21,30^ However, our data demonstrate that SapB accepts the non-phosphorylated **6** as a substrate with high affinity, while no enzymatic activity could be detected with commercially available 2′- or 3′-phosphorylated uridine derivatives (data not shown). Furthermore, the active site of SapH is unlikely to accommodate phosphorylated substrates. Conversion of uridine (**9**) and possibly oxazinomycin (**2**) to their 5′-aminated congeners by SapB and SapH further indicates that early steps in pseudouridimycin biosynthesis are carried out with nucleoside substrates.

## Methods

### Reagents

Pseudouridine was purchased from Jena Bioscience (Jena, Germany). Oxazinomycin was produced in *Streptomyces hygroscopicus* subsp. *hygroscopicus* JCM 4712 and purified as previously described.^18^ All other reagents used were molecular biology grade.

### Bacterial Strains and General DNA Techniques

Codon-optimized synthetic DNA for expression of SapB (GenBank: TGG86068.1) and SapH (Uniprot ID: S3AT34_9ACTN) genes were obtained from Thermo Scientific. The genes were cloned to pBADHisBd plasmids using *Bgl*II and *Hin*dIII restriction enzymes (Thermo Fisher Scientific). Proteins were expressed in *E. coli* TOP10 cells with N-terminal His_6_-tag in 2× TY medium, +30 °C, and 250 rpm. Protein production was induced by adding 0.02% l-arabinose when the *A*_600_ reached 0.8. Cells were incubated at 22 °C, 18 h, and 160 rpm or 30 °C, 140 rpm, and 18 h for the production of SapB and SapH, respectively. Cells were collected by centrifuging for 20 min at 4500*g*.

### Site-Directed Mutagenesis

Mutations were introduced into the SapH gene using specific primers (Thermo Scientific) (Table S2) and Phusion HF polymerase (Thermo Scientific). The mutations were verified by Sanger sequencing (Eurofins Genomics, Germany).

### Protein Purification

Cells were suspended in 20 mL of A-buffer (40 mM Tris-HCl, pH 7.5, 150–300 mM NaCl, 10% glycerol, and 5 mM imidazole) and broken with a French press using 1000 psi pressure. Cell debris was pelleted by centrifuging at 40 000*g* for 40 min at +4 °C. Then, the supernatant was incubated with 1 mL of TALON Sepharose resin for 1 h at +4 °C. Then, the resin was moved to a column and washed twice with 15 mL of buffer A. The protein was eluted with buffer A containing 200 mM imidazole. A PD-10 column was used to remove the imidazole, and the proteins were eluted with a storage buffer (40 mM Tris-HCl, pH 7.5, and 150 mM NaCl). Proteins were concentrated using Amicon 10 kDa cutoff concentrators. Finally, 50% glycerol was added, and the protein preps were stored in a −20 °C freezer. Nanodrop was used to determine the concentrations of the enzymes.

### Enzyme Reactions

The enzymatic reactions were usually incubated for 18 h at +22 °C. A typical reaction mixture included 1 μM SapB, 1 mM pseudouridine, 2 U catalase, 2 μM SapH, 5 mM arginine (or other amino acid), and 20 μM PLP in 25 mM Tris-HCl, pH 7.5, 10 mM NaCl, 10 mM KCl, and 5 mM MgCl_2_ buffer. The reactions were stopped by adding equal volume of CHCl_3_ to precipitate proteins. For the decoupled reactions, SapB was removed by filtering by using an Amicon Ultra 3 kDa molecular weight cut off filter. The water phase was the analyzed with HPLC (SCL-10Avp HPLC with an SPD-M10Avp diode array detector, Shimadzu) or LC/MS (Agilent 6100 Series Quadrupole LC/MS Systems) using a Phenomenex 150 × 4.6 Synergi 4 μm Fusion-RP 80 Å analytical column (flow rate 0.5 mL/min and wavelength 260 nm, 0.1% formic acid in H_2_O and MeCN as eluents). High-resolution electrospray ionization mass spectra were recorded on a Waters ACQUITY RDa detector using a XBridge BEH C18 Column, 130 Å, 5 μm, 4.6 × 30 mm (Waters) using solvent A (H_2_O/0.1% HCOOH) and solvent B (CH_3_OH/0.1% HCOOH) with a flow rate of 0.8 mL/min. The gradient run consisted of 0–2.20 min, 2–100% B; 2.20–2.50 min, 100% B; 2.50–2.80 min, 100–2% B; 2.80–3.00 min, 2% B.

### Cofactor Identification

200 μL of SapB protein (130 μM in C-buffer, no glycerol) was boiled for 10 min. The protein was then centrifuged, and the supernatant was analyzed with HPLC and LC/MS. 50 μM solutions of FAD and flavin mononucleotide were used as references.

### StuB Kinetics

HRP and ABTS were used to follow the H_2_O_2_ formation by measuring *A*_405_ with a Multiskan go plate reader. Reactions were set up in 50 mM Tris-HCl, pH 7.5, 10 mM NaCl, 10 mM KCl, and 5 mM MgCl_2_ in a 100 μL volume. Reactions were initiated by the addition of SapB. Reactions included 0.5–1.5 μM SapB, 1–10 000 μM substrate (uridine, pseudouridine, or oxazinomycin), 0.91 mM ABTS, and 10 ng/mL HRP. The absorbance changes were referenced against H_2_O_2_ standard curve. The slopes of the initial reaction rates were calculated, and the data were fitted to the Michaelis–Menten equation with the OriginLab 8.0 software. Error bars indicate SD of three independent measurements.

### Protein Crystallization and Structure Determination

A solution of recombinant SapH carrying an N-terminal His_6_-tag (MAHHHHHHHRS) in 50 mM tris(hydroxymethyl)methylamino] propanesulfonic acid) (TAPS) buffer, pH 8.5, containing 250 mM NaCl and 10% glycerol at 9.9 mg/mL concentration was used in crystallization screening. Crystals of SapH were obtained at 20 °C using the hanging drop vapor diffusion method in 24-well cell culture plates (Sarstedt, REF: 83.3922, Sarstedt AG & Co. KG, Numbrecht, Germany, EU) by mixing 2 μL of protein solution with 1 μL of the well solution (0.1 M BisTRIS-propane, 0.2 M Na^+^-K^+^-phosphate, and 27.5% PEG3350, pH 7.5). The crystals formed quickly and were picked 6 h after setting up the crystallization drops in a nylon loop (Hampton), flash frozen, and stored in liquid nitrogen until data collection.

An X-ray diffraction data set was collected at the BioMAX beamline^31^ at the MAX-IV synchrotron (Lund, Sweden) at a 1.55 Å resolution. The data set was indexed and integrated using XDS^32^ and scaled with AIMLESS from the CCP4i suite.^33^ The structure was solved by molecular replacement in space group *P*2_1_2_1_2_1_ using MOLREP^34^ and the coordinates of the cofactor free omega transaminase from *Brucella anthropi*, (PDB: 5GHG(35)) as the search model. Model building was carried out in COOT^36^ interspersed by runs of crystallographic refinement in REFMAC-5^37^ employing isotropic *B*-factors. The final model contained two protein chains forming a dimer with one pyridoxal-phosphate cofactor bound per subunit, two glycerol molecules, three potassium ions, and 894 crystallographic water molecules. Model parameters and refinement statistics are summarized in Table 1. The structural model was validated using COOT and MOLPROBITY.^38^ The molecular contacts and interfaces were analyzed using PISA,^39^ structural comparisons were carried out by the DALI algorithm,^40^ and figures were made in PyMOL (www.pymol.org). The final model and the diffraction data were deposited with the Protein Data Bank under accession codes (7QZJ).

## Supporting Information Available

Details of chemical synthesis, protein purification and enzyme reactions, X-ray data, primers, and sequence alignment (PDF)

## Abbreviations

FAD: flavin adenine dinucleotide
PLP: pyridoxal 5′-phosphate
PUM: pseudouridimycin
BGC: biosynthetic gene cluster
HRP: horseradish peroxide
ABTS: 2,2′-azino-bis(3-ethylbenzthiazoline-6-sulfonic acid)
