# Supplementary material for: Pseudouridine-Modifying Enzymes SapB and SapH Control Entry into the Pseudouridimycin Biosynthetic Pathway
Source: ACS Chem Biol. 2023 Apr 3;18(4):794–802. doi: 10.1021/acschembio.2c00826 (PMC10127198; doi:10.1021/acschembio.2c00826)
Supplement: Supplementary file 1 — cb2c00826_si_001.pdf [file cb2c00826_si_001.pdf]

Supplementary Information

The pseudouridine modifying enzymes SapB and SapH control entry into the pseudouridimycin biosynthetic pathway

Erika Artukka[1], Robert Schnell[2], Kaisa Palmu[1], Petja Rosenqvist[3], Edit Szodorai[4], Jarmo Niemi[1], Pasi Virta[3], Gunter Schneider[4], and Mikko Metsä-Ketelä[1,*]

[1] Department of Life Technologies and [3] Department of Chemistry, University of Turku, Turku, FIN-20014 Turku, Finland

[2] Department of Neuroscience and [4] Department of Medical Biochemistry and Biophysics, Karolinska Institutet, SE-17177 Stockholm, Sweden

[*]Correspondence: mianme@utu.fi

Supporting information text

Synthesis of 5´-amino-5´-deoxyuridine

Uridine (0.20 g, 0.82 mmol) and dry triethylamine (0.34 ml, 2.5 mmol) were dissolved in dry pyridine (3.4 ml) and the solution was mixed at 0 °C for 5 minutes under nitrogen. Then, *p*-toluenesulfonyl chloride (0.16 g, 0.82 mmol) was added and the reaction was stirred for two days at room temperature. The reaction solution was diluted with ethyl acetate (10 ml), washed with saturated NH_4Cl (5 ml), water (5 ml) and saturated NaCl (10 ml), dried over Na_2SO_4, filtered, and evaporated under reduced pressure. The obtained crude 5'-O-*p*-tolueneosulfonylated uridine was co-evaporated three times with dry toluene and dissolved in dry DMF (2.2 ml). NaN_3 (0.43 g, 6.6 mmol) was added and the mixture was stirred at 60 °C for 48 hours, and then concentrated under reduced pressure. The residue was passed through a short silica gel column by eluting with 10 % MeOH in DCM. The fractions of 5'-azido-5'-deoxyuridine were evaporated to dryness under vacuum. The residue and triphenylphosphine (50 mg, 0.19 mmol) were dissolved in MeCN/H_2O/THF (4.0 ml: 0.8 ml: 3.0 ml), and the mixture was stirred at room temperature for 18 hours. Water (6 ml) was added and the mixture was washed two times with ethyl acetate (4 ml). The aqueous phase was concentrated under vacuum and the crude product was purified by RP-HPLC (Thermo Scientific 250 x 4 ODS HYPERSIL 5 µm analytical column) eluting with water. The product fractions were combined and lyophilized to yield 5´-amino-5´-deoxyuridine as white solid (12 mg, 6 %). ^{1}H NMR δ_H (500 MHz, D_2O): 7.59 (d, 1H, *J* = 8.1 Hz, H-6), 5.82 (d, 1H, *J* = 8.1 Hz, H-5), 5.69 (d, 1H, *J* = 4.0 Hz, H-1´) 4.45 (dd, 1H, *J* = 4.0 and 5.0 Hz, H-2´), 4.19 – 4.15 (m, 2H, H-3´ & H-4´), 3.41 – 3.38 (m, 1H, H-5´), 3.29 – 3.22 (m, 1H, H-5´´) (Figure S3); ^{13}C NMR δ_C (125 MHz, CD_3OD): 166.2 (C-4), 151.4 (C-2), 143.2 (C-6), 102.2 (C-5), 92.7 (C-1´), 79.3 (C-4´), 72.6 (C-2´), 70.8 (C-3´), 41.2 (C-5´) (Figure S4). HRMS (ESI) *m/z*: $[M+H]^+$ calculated for $C_9H_{14}N_3O_5^+$ 244.0928; found 244.0934 (Figure S5).

Sequence analysis and alignment of proteins from the glucose-methanol-choline oxidoreductase family.

The SapB protein sequence was used as query in BLAST search against pdb. After manual dereplication of identical sequences, glucose-methanol-choline oxidoreductases were aligned using Clustal W Multiple Alignment tool in BioEdit software. The retrieved sequences that were used were: pdb|4UDP|A 5-hydroxymethylfurfural oxidase [*Methylovorus* sp. MP688]; pdb|3T37|A pyridoxine 4-oxidase [*Mesorhizobium loti*]; pdb|3LJP|A Choline oxidase [*Arthrobacter globiformis*]; pdb|6YS1|AAA Chain AAA, Fatty acid Photodecarboxylase [*Chlorella variabilis*]; pdb|4YNT|A FAD glucose dehydrogenase [*Aspergillus flavus* NRRL3357]; pdb|1CF3|A glucose oxidase [*Aspergillus niger*]; pdb|3Q9T|A formate oxidase [*Aspergillus oryzae* RIB40]; pdb|6ZE2|A FADdependent oxidoreductase [*Thermochaetoides thermophila* DSM 1495]; pdb|3FIM|B arylalcoholoxidase [*Pleurotus eryngii*]; pdb|6H3O|A Alcohol oxidase [*Phanerodontia chrysosporium*]; pdb|1JU2|A hydroxynitrile lyase from almond [*Prunus dulcis*]; pdb|4QI4|A

Cellobiose dehydrogenase [*Thermothelomyces myriococcoides*]; pdb|1NAA|A Cellobiose dehydrogenase [*Phanerodontia chrysosporium*].

Supplementary figures

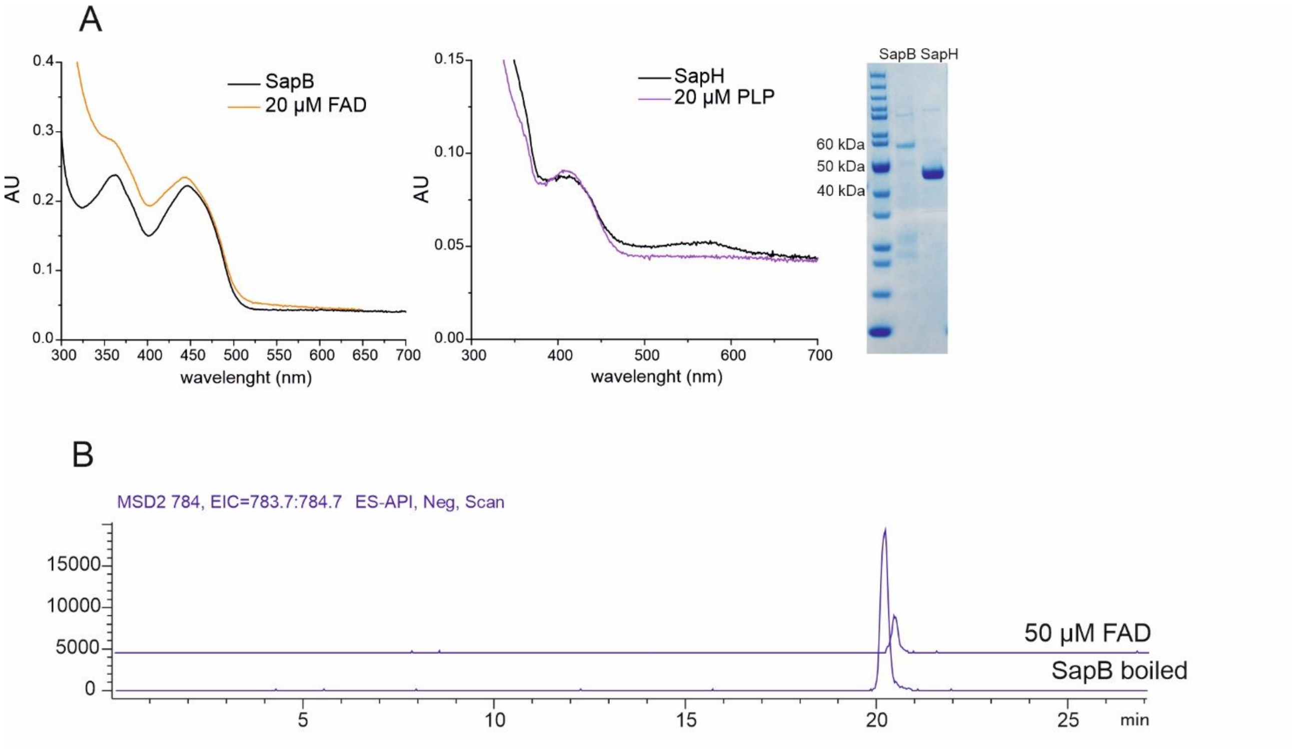

Figure S1. Characterization of SapB and SapH cofactors. a) UV/Vis spectra of SapB and SapH. SDS-PAGE gel of the purified proteins. b) Verification of the cofactor of SapB as FAD. SapB protein was boiled 10 min. After centrifugation the supernatant was analyzed with LC/MS and compared to FAD standard.

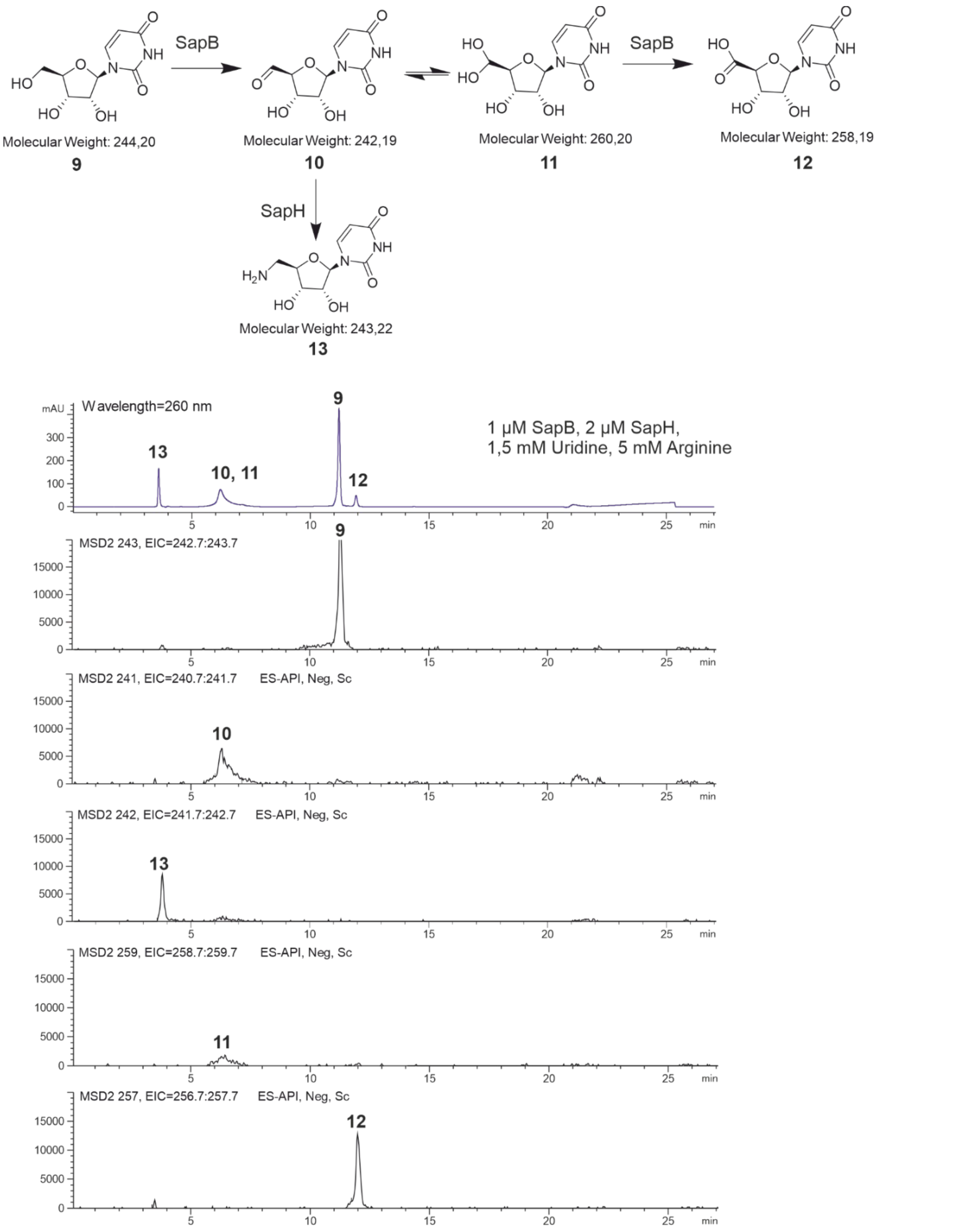

Figure S2. Analysis of SapB and SapH reactions by LC/MS. Diol and carboxylic acid derivatives (top) may be formed as shunt products during the reaction. Identification of compounds (bottom) based on UV-Vis spectrum and extracted ion chromatograms of SapB and SapH reaction with uridine **9**.

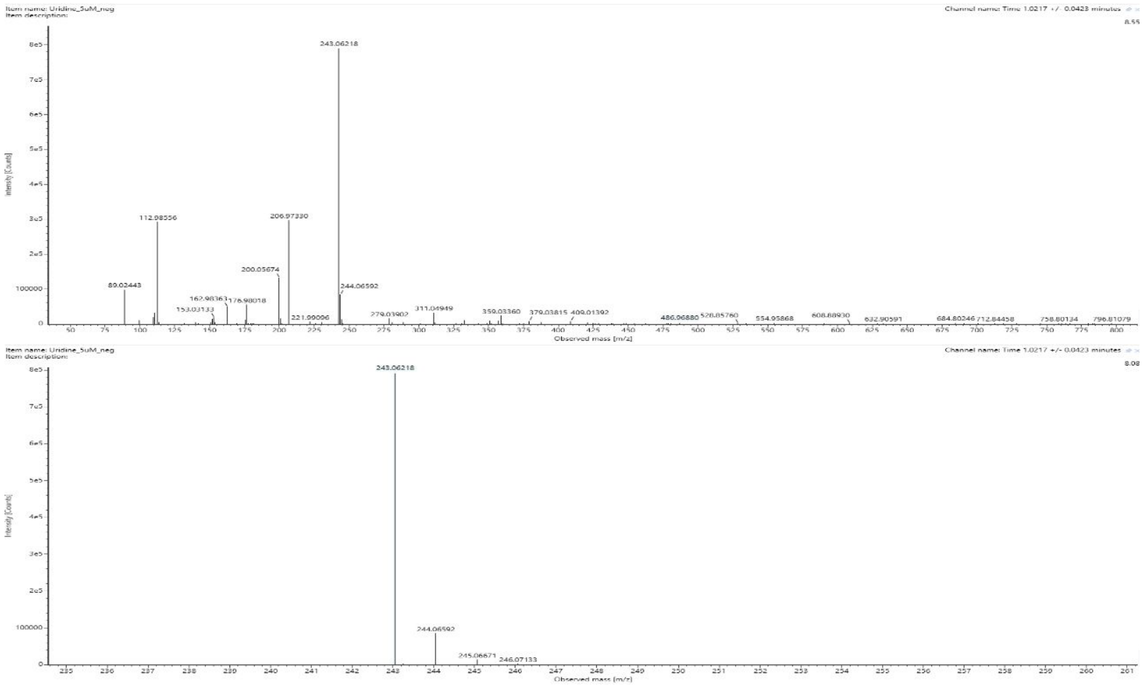

Figure S3. (-)-HRESI-MS spectrum of substrate 9. Calculated [M-H]- = 243.0622, measured [M-H]- = 243.0622.

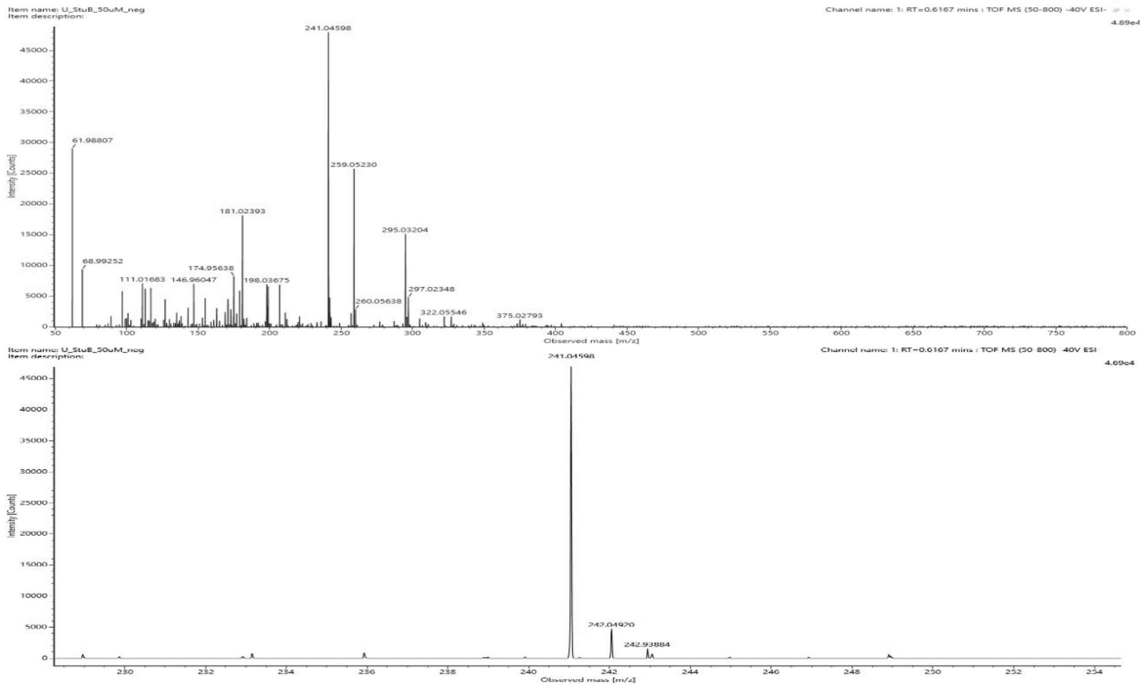

Figure S4. (-)-HRESI-MS spectrum of 10 acquired from the enzymatic reaction of 9 with SapB. Calculated [M-H]- = 241.0466, measured [M-H]- = 241.0460.

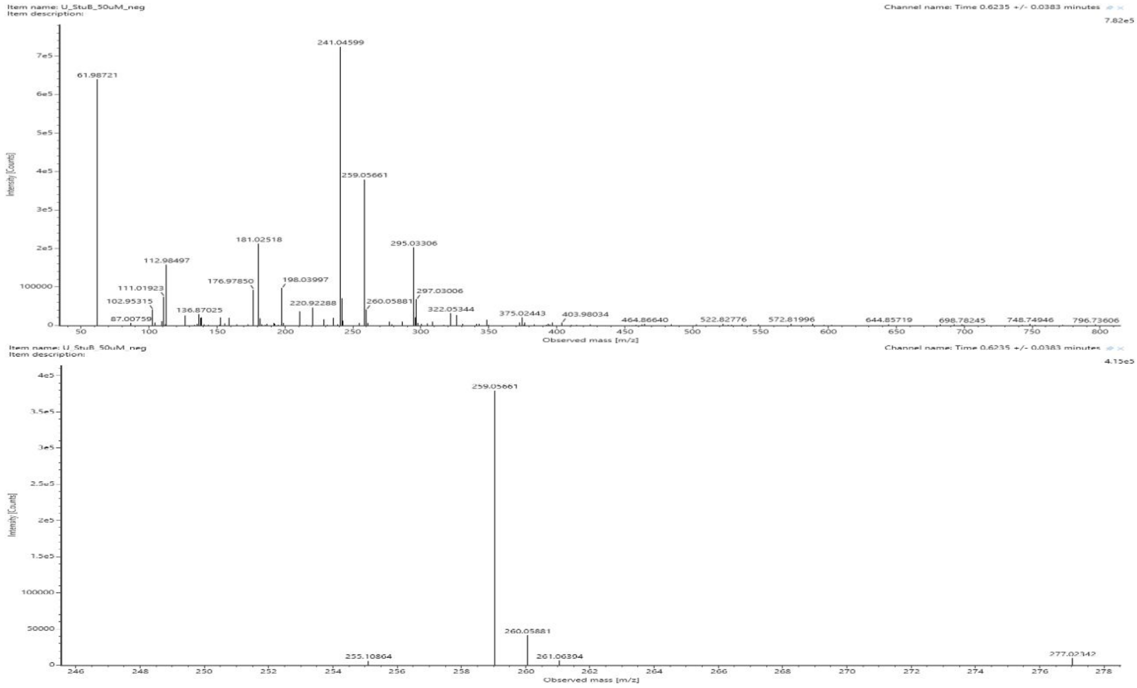

Figure S5. (-)-HRESI-MS spectrum of 11 acquired from the enzymatic reaction of 9 with SapB. Calculated [M-H]- = 259.0572, measured [M-H]- = 259.0566.

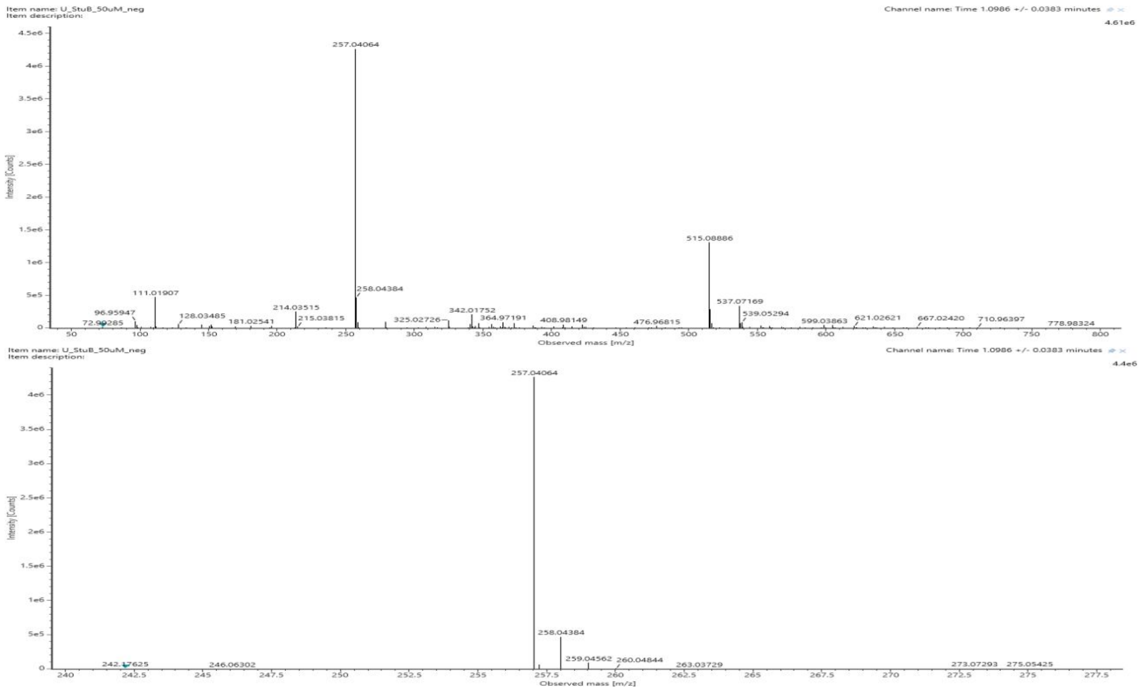

Figure S6. (-)-HRESI-MS spectrum of 12 acquired from the enzymatic reaction of 9 with SapB. Calculated [M-H]- = 257.0415, measured [M-H]- = 257.0406.

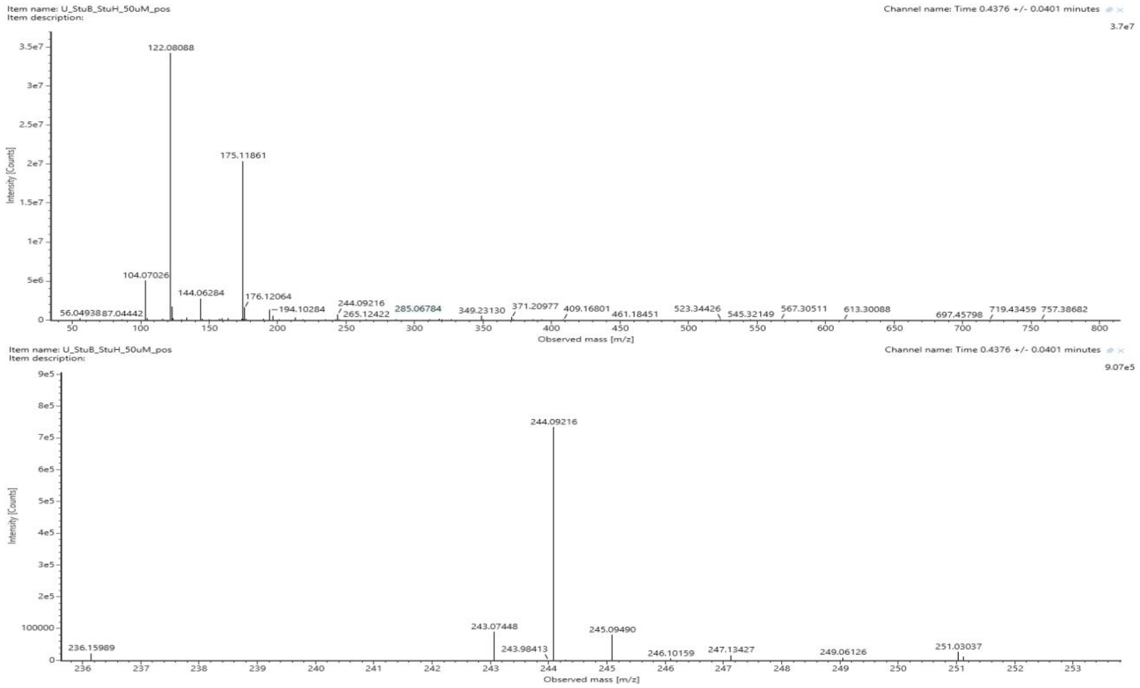

Figure S7. (+)-HRESI-MS spectrum of 13 acquired from the enzymatic reaction of 9 with SapB+SapH. Calculated [M+H]+ = 244.0928, measured [M+H]+ = 244.0922.

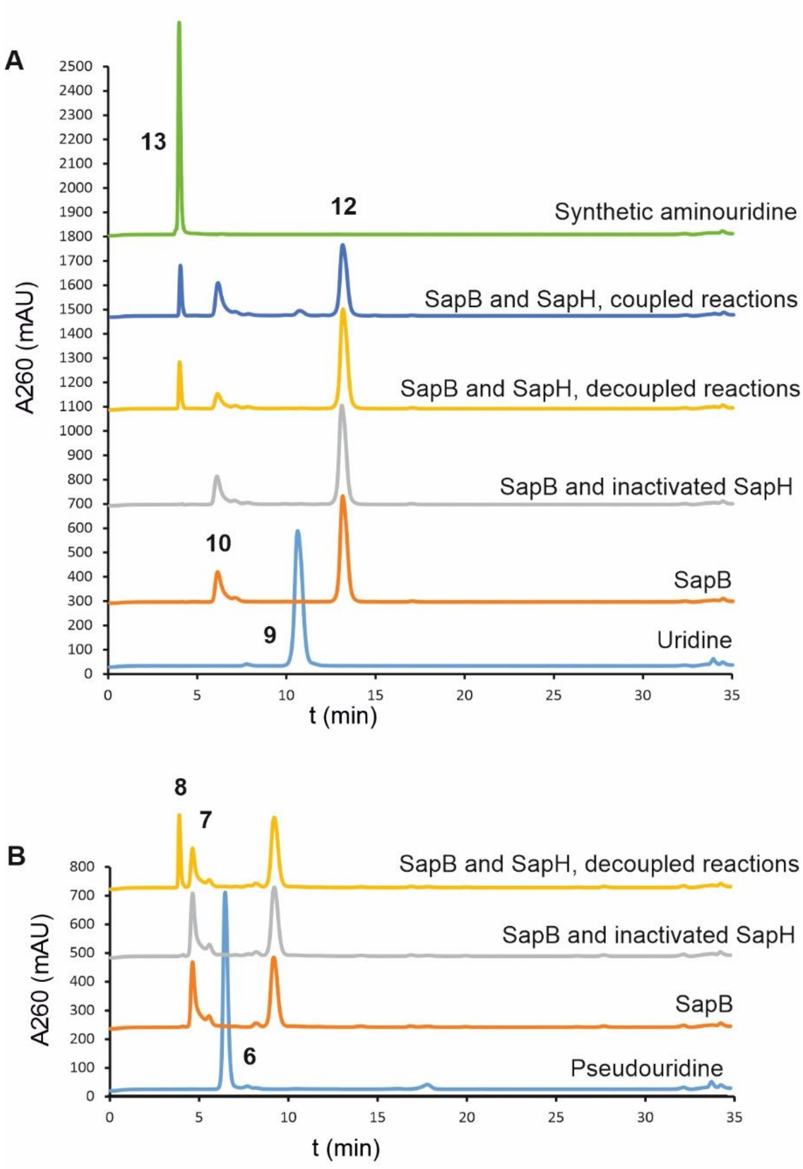

Figure S8. Separation of SapB and SapH reactions. a) HPLC data indicating the conversion of **9** to **13**. The chromatogram traces depict from top to bottom: positive control for **13**; a coupled reaction of SapB and SapH where enzymes have been incubated with **9**; a decoupled reaction where SapB was first incubated with **9** to produce **10**, followed by filtration of sample to remove enzyme and addition of SapH to show further conversion to **13**; a control for the decoupled reaction where inactivated SapH has been added to demonstrate lack of conversion of **10** to **13**; a control reaction demonstrating conversion of **9** to **10** by SapB; negative control **9**. b) HPLC data indicating the initial conversion of **6** to **7** by SapB, and further conversion of **7** to **8** by SapH after removal of SapB by filtration. SapH was inactivated by boiling in the control reaction.

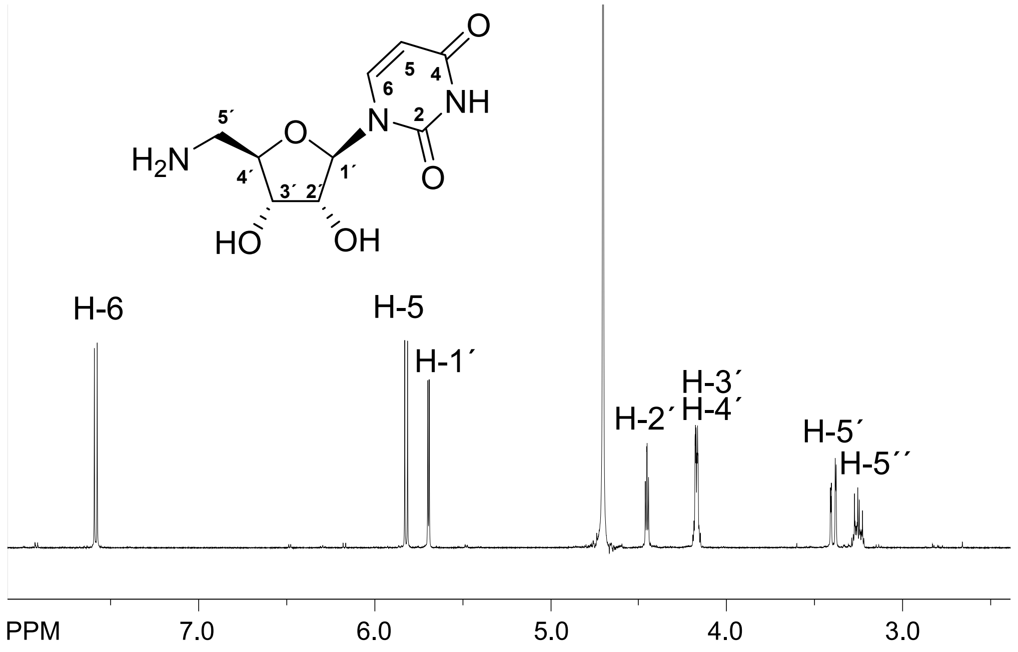

Figure S9. ^{1}H NMR (500 MHz, D_2O) spectrum of 5´-amino-5´-deoxyuridine **13**.

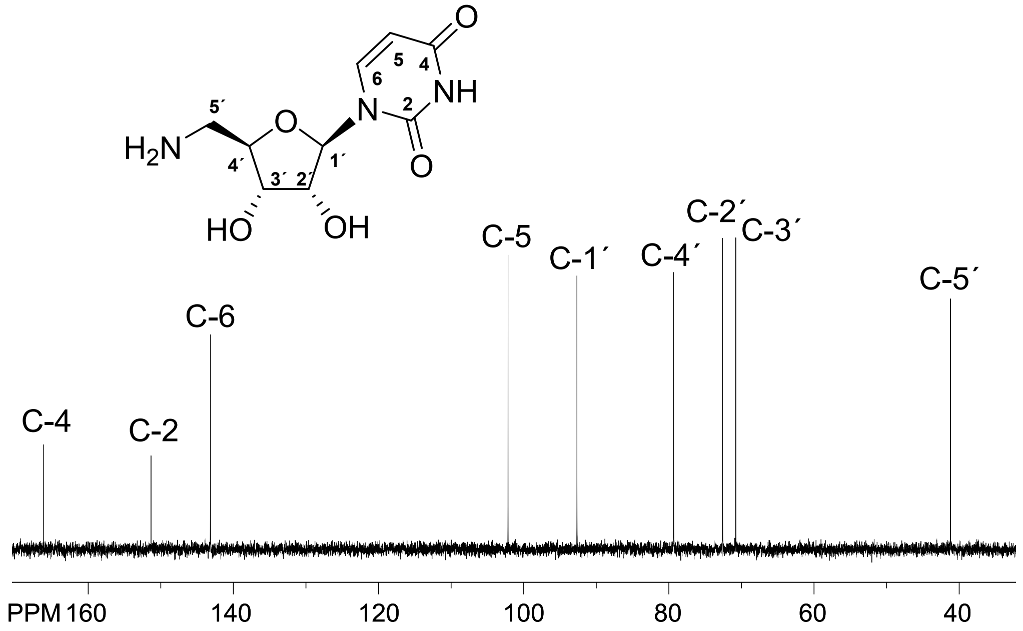

Figure S10. ^{13}C NMR (125 MHz, D_2O) spectrum of 5´-amino-5´-deoxyuridine **13**.

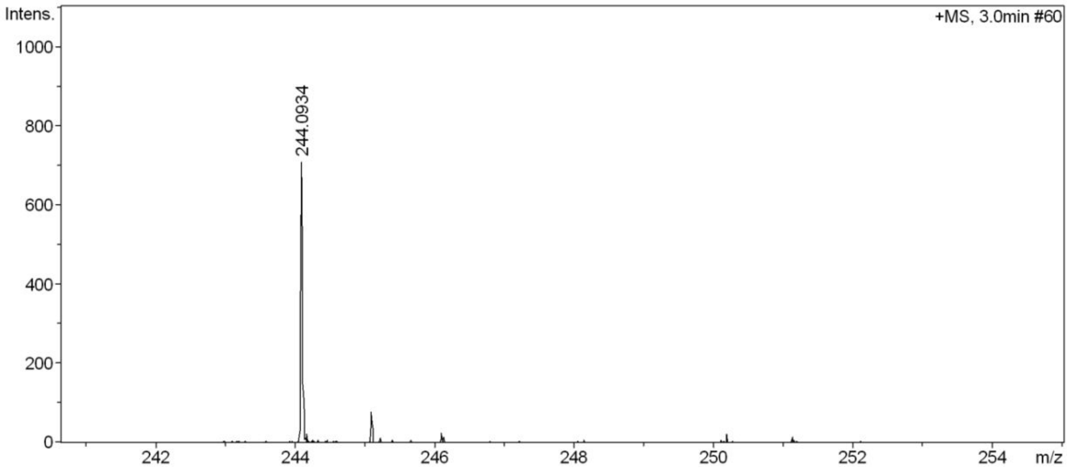

Figure S11. (+)-HRESI-MS spectrum of synthetic 5´-amino-5´-deoxyuridine **13**.

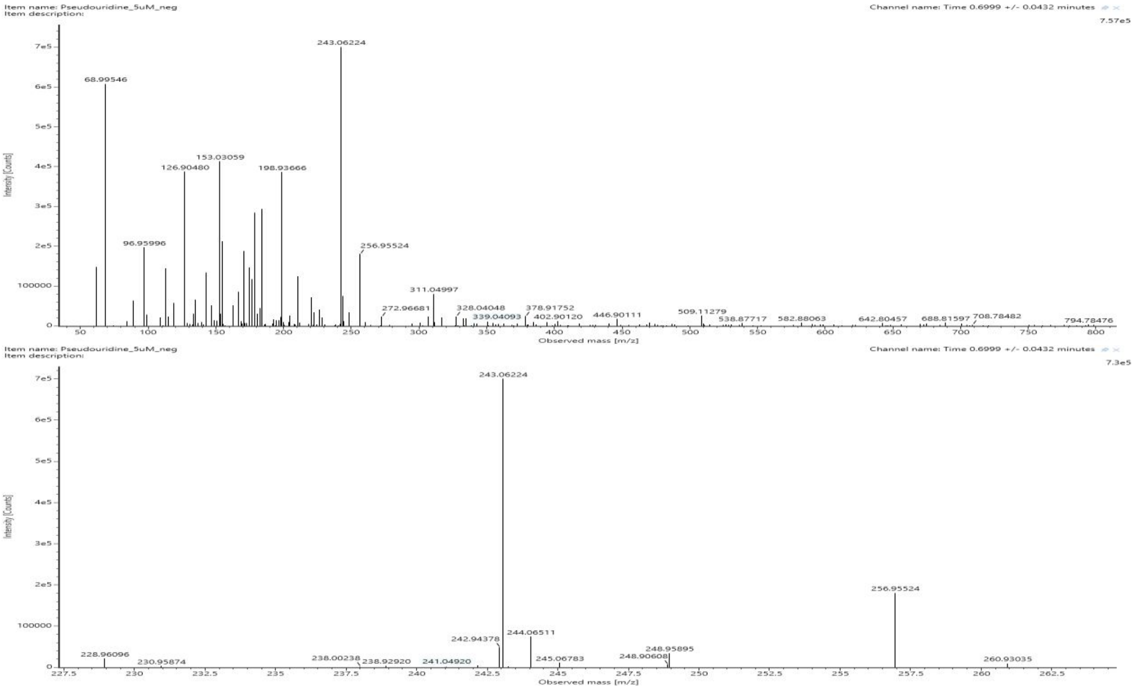

Figure S12. (-)-HRESI-MS spectrum of substrate **6**. Calculated [M-H]- = 243.0622, measured [M-H]- = 243.0622.

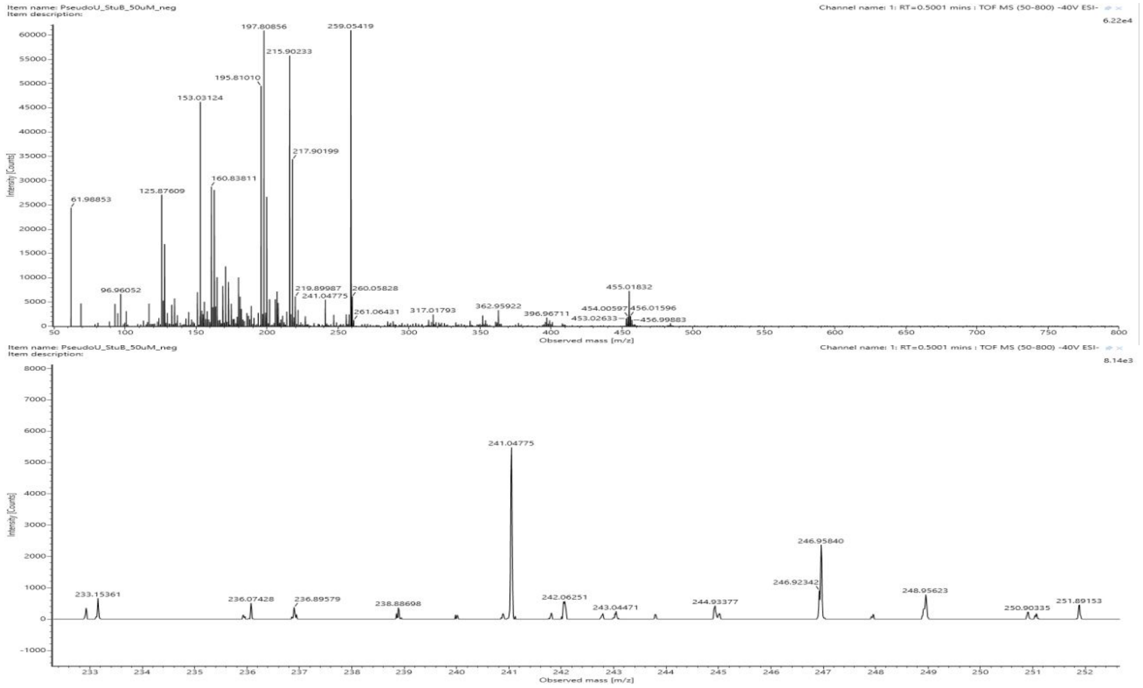

Figure S13. (-)-HRESI-MS spectrum of **7** acquired from the enzymatic reaction of **6** with SapB. Calculated [M-H]- = 241.0466, measured [M-H]- = 241.0478.

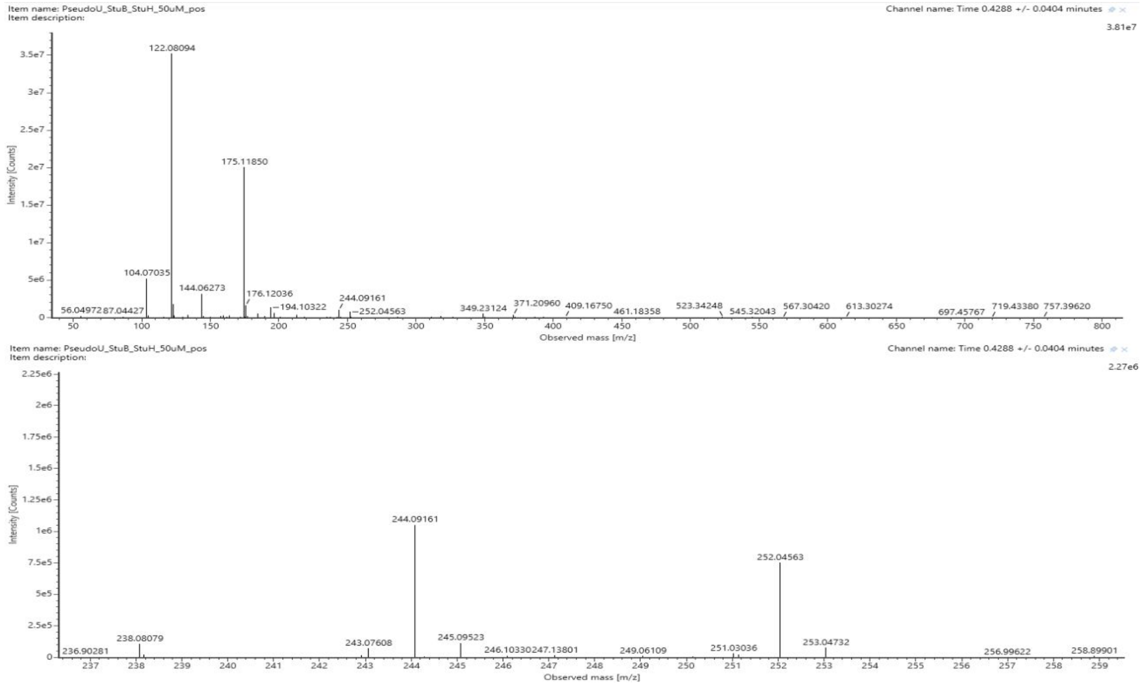

Figure S14. (+)-HRESI-MS spectrum of **8** acquired from the enzymatic reaction of **6** with SapB+SapH. Calculated [M+H]+ = 244.0928, measured [M+H]+ = 244.0922.

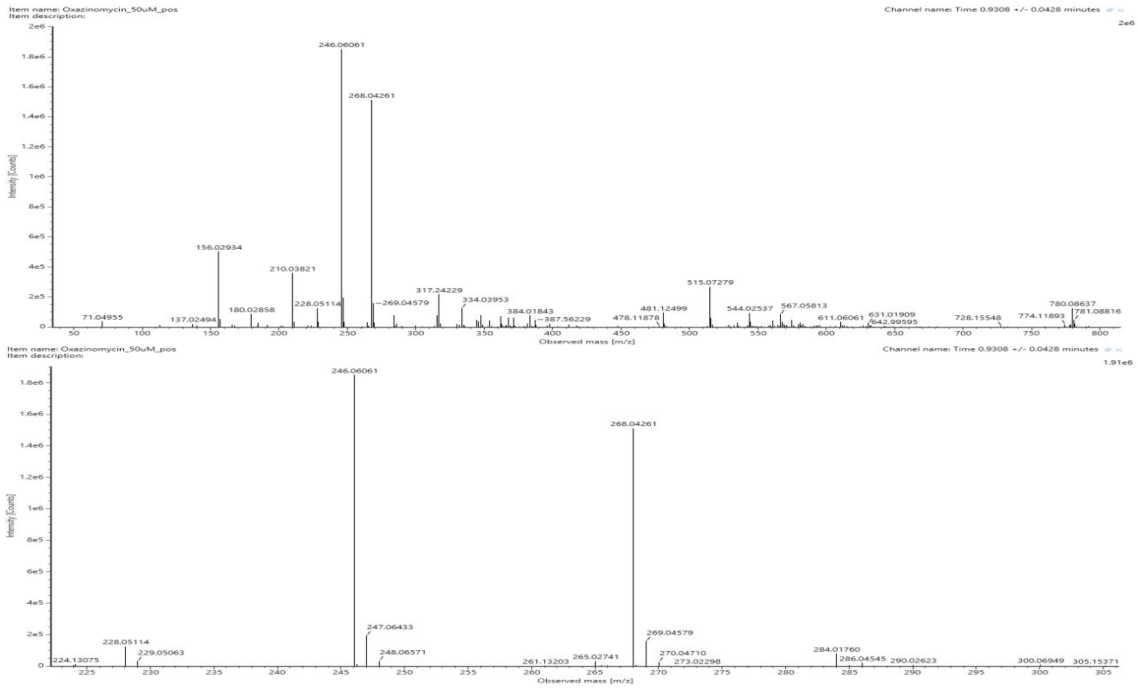

Figure S15. (+)-HRESI-MS spectrum of substrate **2**. Calculated [M+H]+ = 246.0608, measured [M+H]+ = 246.0606.

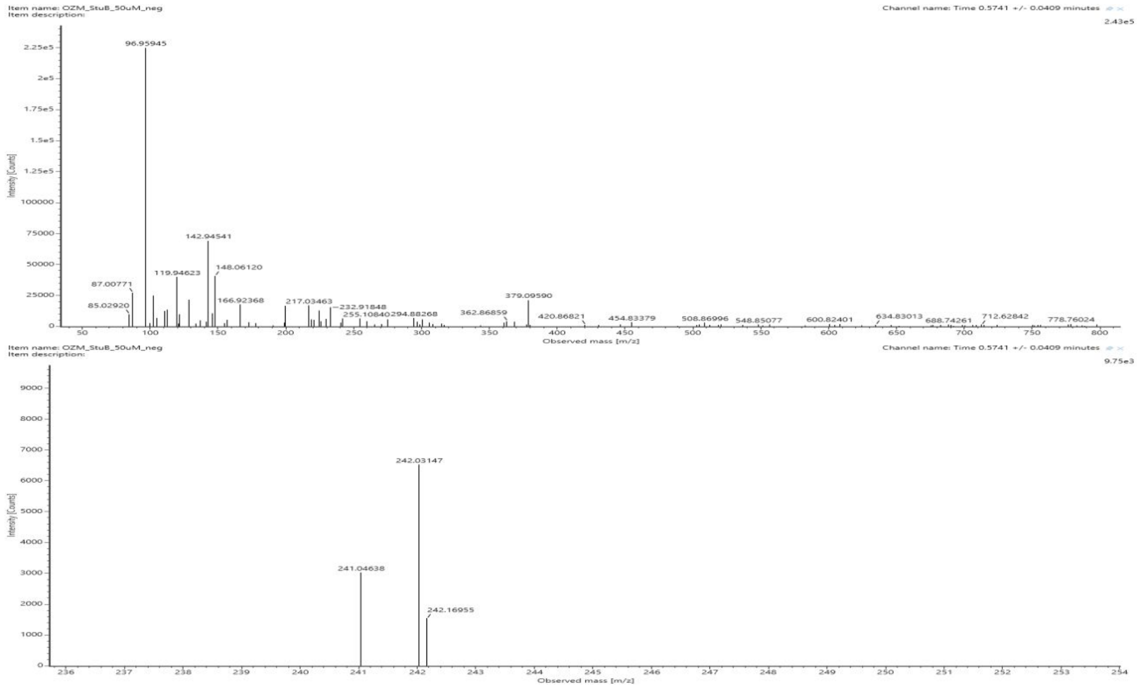

Figure S16. (-)-HRESI-MS spectrum of **14** acquired from the enzymatic reaction of **2** with SapB. Calculated [M-H]- = 242.0306, measured [M-H]- = 242.0315.

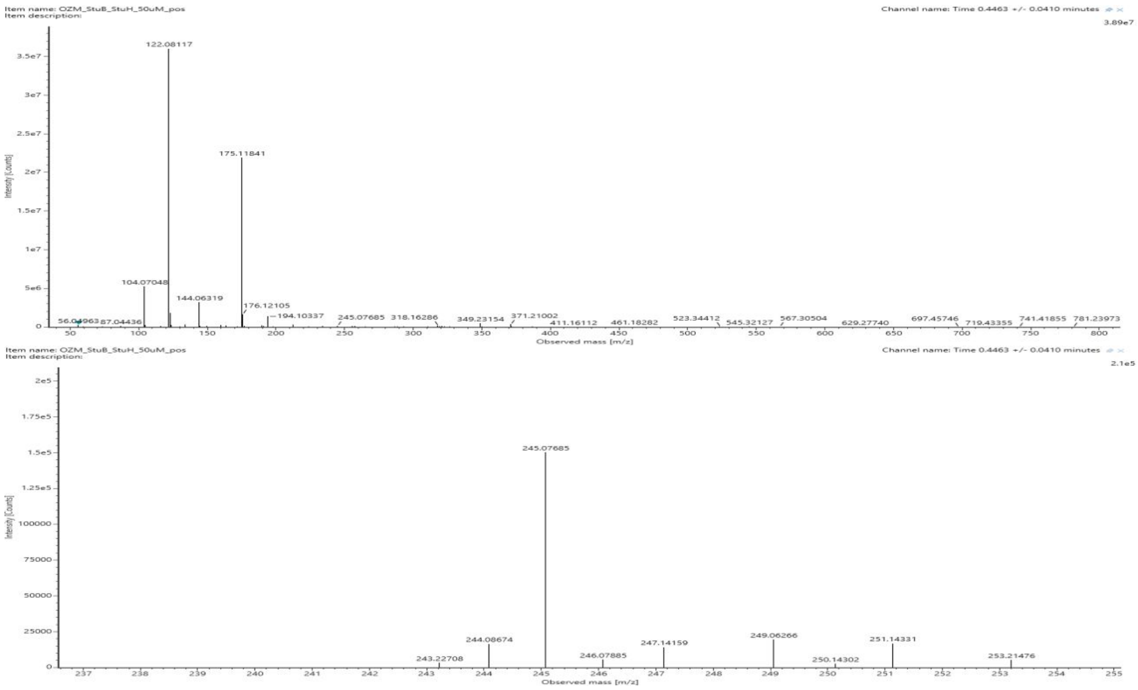

Figure S17. (+)-HRESI-MS spectrum of **15** acquired from the enzymatic reaction of **2** with SapB+SapH. Calculated [M+H]+ = 245.0768, measured [M+H]+ = 245.0769.

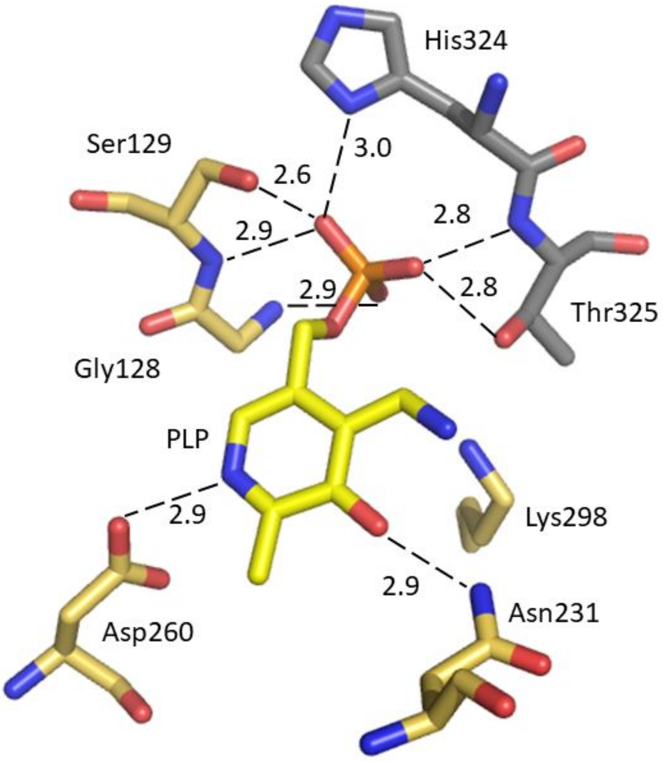

Figure S18. The PLP cofactor bound at the dimer interface is coordinated by residues from both protein chains forming the dimer. The pyridoxal-amine form of the cofactor observed in the crystal structure is

depicted as sticks with yellow carbons, the invariant residue Lysine-289 that is expected to form the internal aldimine in the ground state of the enzyme is shown next to its binding position. Amino acid residues involved in PLPL coordination that form hydrogen bonds are displayed in beige (one of the chains) or in gray (the second chain of the dimer). Dashed lines indicate potential hydrogen bonds with the distances indicated in Å.

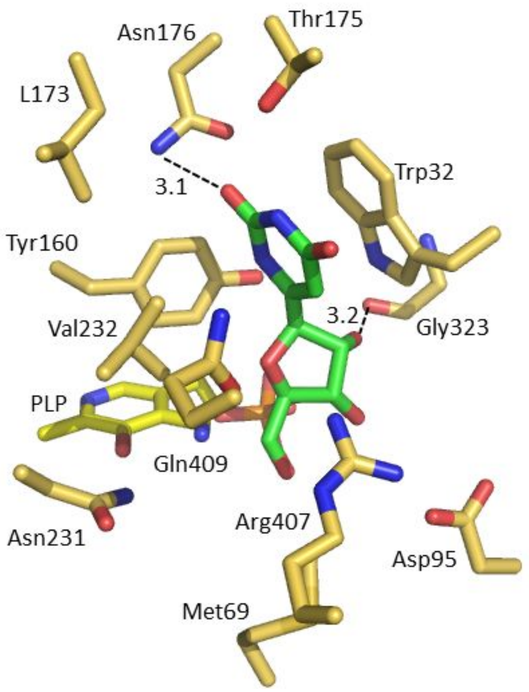

Figure S19. Stereo view image of the putative binding mode of **7** in the active site of SapH based on manual docking.

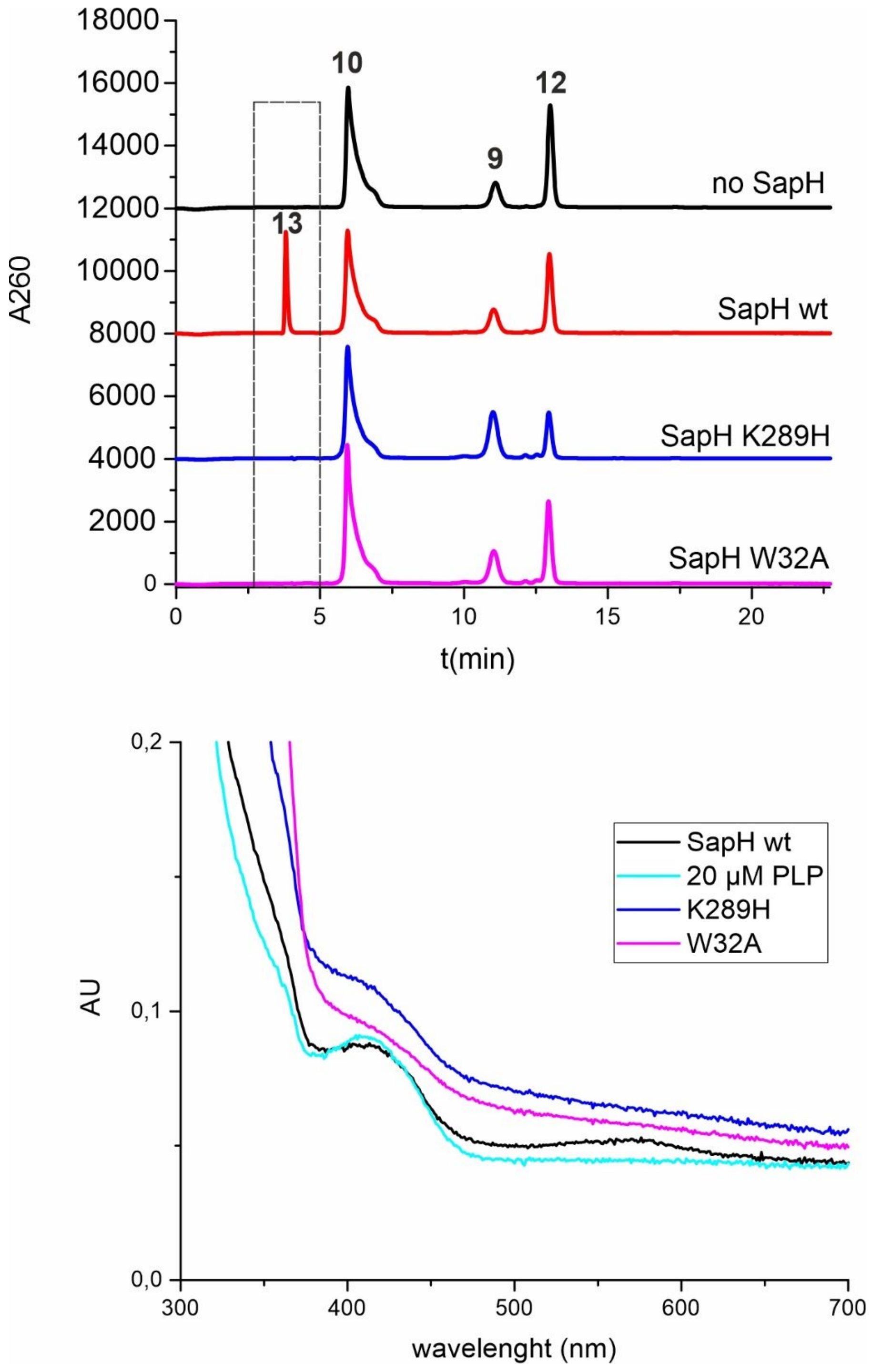

Figure S20. **Analysis of SapH K289H and W32A variant enzymes.** HPLC data (top) demonstrates that SapH K289H and W32A are inactive and no product **13** formation can be observed in coupled reactions with SapB and **9** as a substrate. Analysis of UV/Vis spectrum (bottom) of purified SapH K289H and W32A proteins demonstrates lack of an absorption maximum typical for PLP-dependent enzymes.

```
                     10        20        30        40        50        60
            ....|....|....|....|....|....|....|....|....|....|....|....|
Query SapB  ----------------------MPHGFDDIVVGAGSAGAVLAARLS-ADPGRRVLLVEAG 37
pdb|4UDP|   ---------------------MTDTIFDYVIVGGGTAGSVLANRLS-ARPENRVLLIEAG 38
pdb|3T37|   ---------MADGVRMTRAKVEHAPNCDIVIVGGGSAGSLLAARLS-EDPDSRVLLIEAG 50
pdb|3LJP|   -------------MHIDNIENLSDREFDYIVVGGGSAGAAVAARLS-EDPAVSVALVEAG 46
pdb|4YNT|   ---------------------MNTTTYDYIVVGGGTSGLVVANRLS-ENPDVSVLLLEAG 38
pdb|1CF3|   -------SNGIEASLLTDPKDVSGRTVDYIIAGGGLTGLTTAARLT-ENPNISVLVIESG 52
pdb|3Q9T|   --------------------ATDGSHFDFVIVGGGTAGNTVAGRLA-ENPNVTVLIVEAG 39
pdb|3FIM|   ------------------------ADFDYVVVGAGNAGNVVAARLT-EDPDVSVLVLEAG 35
pdb|6H3O|   --------------------MGHPEEVDVIVCGGGPAGCVVAGRLAYADPTLKVMLIEGG 40
pdb|1JU2|   LATTSDHDFSYLSFAYDATDLELEGSYDYVIVGGGTSGCPLAATLS---EKYKVLVLERG 57

                     70        80        90       100       110       120
            ....|....|....|....|....|....|....|....|....|....|....|....|
Query SapB  ---PDPD--PAALPADLRDGCTPSMVDHDWGLEGIRDDGARLPLPR-------GRVVGGS 85
pdb|4UDP|   IDTPENNIPPEIHDGLRPWLPRLSGDKFFWPNLTIHRAAEHPGITREPQFYEQGRLLGGG 98
pdb|3T37|   ----EEPTDPDIWNPAAWPALQGRSYDWDYRTEAQAGTAGRAHHWAR------GRLIGGS 100
pdb|3LJP|   ---PDDRGVPEVLQLDRWMELLESGYDWDYPIEPQENGNSFMRHAR-------AKVMGGC 96
pdb|4YNT|   A--SVFN-NPDVTNANGYGLAFGSAIDWQYQSINQSYAGGKQQVLRA------GKALGGT 89
pdb|1CF3|   S--YESDRGPIIEDLNAYGDIFGSSVDHAYETVELATNN-QTALIRS------GNGLGGS 103
pdb|3Q9T|   IG--NPEDIPEITTPSSAMDLRNSKYDWAYKTTMVRRDDYERIEKPN----TRGKTLGGS 93
pdb|3FIM|   VS--DENVLGAEAPLLAPGLVPNSIFDWNYTTTAQAGYNGRSIAYPR------GRMLGGS 87
pdb|6H3O|   ANNRDDPWVYRPGIYVRNMQRNGINDKATFYTDTMASSYLRGRRSIVP----CANILGGG 96
pdb|1JU2|   S---LPTAYPNVLTADGFVYNLQQEDDGKTPVERFVSEDGIDNVRGR--------VLGGT 106

                    130       140       150       160       170       180
            ....|....|....|....|....|....|....|....|....|....|....|....|
Query SapB  SAVNSCIALRPAPGDFAAWDEITGG-DWSWPHMLPWFRRLETDTDFS-----------GP 133
pdb|4UDP|   SSVNMVVSNRGLPRDYDEWQ-ALGADGWDWQGVLPYFIKTERDADYGD----------DP 147
pdb|3T37|   SCLHAMGYMRGHPSDFQAWVDASGDRRWGWDELLPVFQAIEDHPLGGD-----------G 149
pdb|3LJP|   SSHNSCIAFWAPREDLDEWEAKYGATGWNAEAAWPLYKRLETNEDAGP---------DAP 147
pdb|4YNT|   STINGMAYTRAEDVQIDVWQ-KLGNEGWTWKDLLPYYLKSENLTAPTSSQVAAGAAYNPA 148
pdb|1CF3|   TLVNGGTWTRPHKAQVDSWETVFGNEGWNWDNVAAYSLQAERARAPNAKQIAAGHYFNAS 163
pdb|3Q9T|   SSLNYFTWVPGHKATFDQWE-EFGGKEWTWDPLVPYLRKSATYHDDPRLYSP-----ELE 147
pdb|3FIM|   SSVHYMVMMRGSTEDFDRYAAVTGDEGWNWDNIQQFVRKNEMVVPPADNHNTSG-EFIPA 146
pdb|6H3O|   SSINSQMYTRASASDWDDFK----TEGWTCKDLLPLMKRLENYQKPCN----------ND 142
pdb|1JU2|   SIINAGVYARANTSIYSASG-----VDWDMDLVNQTYEWVEDTIVYKP------------ 149

                    190       200       210       220       230       240
            ....|....|....|....|....|....|....|....|....|....|....|....|
Query SapB  VHGTDGPVRLR-RCTGNGLSPLSAALVETATAAGHPYTADHN-APDATGVGPLPLNLTED 191
pdb|4UDP|   LHGNAGPIPIG-RVDSRHWSDFTVAATQALEAAGLPNIHDQN---ARFDDGYFPPAFTLK 203
pdb|3T37|   IHGKGGPLPIH--LPADEVSPLARAFIEAGASLGLPRLEGHN---SGEMIGVTPNSLNIR 204
pdb|3LJP|   HHGDSGPVHLM-NVPPK--DPTGVALLDACEQAGIPRAKFNTGTTVVNGANFFQINRRAD 204
pdb|4YNT|   VNGKEGPLKVG-WSGSLASGNLSVALNRTFQAAGVPWVEDVN-GGKMRGFNIYPSTLDVD 206
pdb|1CF3|   CHGVNGTVHAGPRDTGDDYSPIVKALMSAVEDRGVPTKKDFG-CGDPHGVSMFPNTLHED 222
pdb|3Q9T|   KIGGGGPIPISHAELIDEMAPFRENLTKAWKSMGQPLIENIY------DGEMDGLTHCCD 201
pdb|3FIM|   VHGTNGSVSIS---LPGFPTPLDDRVLATTQEQSEEFFFNPD-MGTGHPLGISWSIASVG 202
pdb|6H3O|   THGYDGPIAIS---NGGQIMPVAQDFLRAAHAIGVPYSDDIQDLTTAHGAEIWAKYINRH 199
pdb|1JU2|   -----------------NSQSWQSVTKTAFLEAGVHPNHGFS---LDHEEGTRITGSTFD 189

                    250       260       270       280       290       300
            ....|....|....|....|....|....|....|....|....|....|....|....|
Query SapB  G-TRISAALAYLVPAR-SRPNLTVLAGTLADRVLFSGS-TATGVLLRS---------GSR 239
pdb|4UDP|   GEERFSAARGYLDASVRVRPNLSLWTESRVLKLLTTG-NAITGVSVLR---------GRE 253
pdb|3T37|   DGRRVTAADAWLTKAVRGRKNLTILTGSRVRRLKLEGNQVRSLEVVGR----------QG 254
pdb|3LJP|   G-TRSSSSVSYIHPIV-EQENFTLLTGLRARQLVFDADRRCTGVDIVDSA------FGHT 256
pdb|4YNT|   LNVREDAARAYYFPYD-DRKNLHLLENTTANRLFWKNGSAEEAIADGVEITSA---DGKV 262
pdb|1CF3|   Q-VRSDAAREWLLPNY-QRPNLQVLTGQYVGKVLLSQ-NGTTPRAVGVEFGTH---KGNT 276
pdb|3Q9T|   TIYRGQRSGSFLFVKN--KPNITIVPEVHSKRLIINEADRTCKGVTVVTAAG------NE 253
```

```
pdb|3FIM|     NGQRSSSSTAYLRPAQ-SRPNLSVLINAQVTKLVNSGTTNGLPAFRCVEYAEQE--GAPT 259
pdb|6H3O|     TGRRSDAATAYVHSVMDVQDNLFLRCNARVSRVLFDDNNKAVGVAYVPSRNRTHGGKLHE 259
pdb|1JU2|     NKGTRHAADELLNKGN--SNNLRVGVHASVEKIIFSNAPGLTATGVIYRDSNG---TPHQ 244

                     310       320       330       340       350       360
              ....|....|....|....|....|....|....|....|....|....|....|....|
Query SapB    TRTVHADRVTLCAGAYGTPALLHRSGIGPRPVLDALGVPPVAELTGVGAQLADHAQVPIG 299
pdb|4UDP|     TLQVQAREVILTAGALQSPAILLRTGIGPAADLHALGIPVLADRPGVGRNLWEHSSIGVV 313
pdb|3T37|     SAEVFADQIVLCAGALESPALLMRSGIGPHDVLDAAGVGCLIDMPDIGRNLQDHLLGAGN 314
pdb|3LJP|     HRLTARNEVVLSTGAIDTPKLLMLSGIGPAAHLAEHGIEVLVDSPGVGEHLQDHPEGVVQ 316
pdb|4YNT|     TRVHAKKEVIISAGALRSPLILELSGVGNPTILKKNNITPRVDLPTVGENLQDQFNNGMA 322
pdb|1CF3|     HNVYAKHEVLLAAGSAVSPTILEYSGIGMKSILEPLGIDTVVDLP-VGLNLQDQTTATVR 335
pdb|3Q9T|     LNFFADREVILSQGVFETPKLLMLSGIGPTRELSRHGINTIVDSRHVGQNLMDHPGVPFV 313
pdb|3FIM|     TTVCAKKEVVLSAGSVGTPILLQLSGIGDENDLSSVGIDTIVNNPSVGRNLSDHLLLPAA 319
pdb|6H3O|     TIVKARKMVVLSSGTLGTPQILERSGVGNGELLRQLGIKIVSDLPGVGEQYQDHYTTLSI 319
pdb|1JU2|     AFVRSKGEVIVSAGTIGTPQLLLLSGVGPESYLSSLNIPVVLSHPYVGQFLHDNPRNFIN 304

                     370       380       390       400       410       420
              ....|....|....|....|....|....|....|....|....|....|....|....|
Query SapB    VLPVPGLCDPADPCA--QVVLRCTAPGSSV------------------------------ 327
pdb|4UDP|     APLTEQARADASTGK--AGSRHQLGIRASSG----------------------------- 342
pdb|3T37|     LYAARKPVPPSRLQH--SESMAYMRADSFTAAG--------------------------- 345
pdb|3LJP|     FEAKQPMVAESTQWW--EIGIFTPTEDGLD------------------------------ 344
pdb|4YNT|     GEGYGVLAG-ASTVT--YPSISDVFGNETDSIVASLR-------------SQLSDYAAAT 366
pdb|1CF3|     SRITSAGAGQGQAAW--FATFNETFGDYSEKAHELLN-------------TKLEQWAEEA 380
pdb|3Q9T|     LRVKDGFGMDDVLLR--HGPKRDAVVSAYNKNRSGPVGSGLLELVGFPRIDKYLEKDAEY 371
pdb|3FIM|     FFVNSNQTFDNIFRD--SSEFNVDLDQWTNTRTGPLT-------------ALIANHLAWL 364
pdb|6H3O|     YRVSNESITTDDFLRGVKDVQRELFTEWEVSPEKARLSSNAIDAG-----FKIRPTEEEL 374
pdb|1JU2|     ILPPNPIEPTIVTVLGISNDFYQCSFSSLP------------------------------ 334

                     430       440       450       460       470       480
              ....|....|....|....|....|....|....|....|....|....|....|....|
Query SapB    ---------------ADDLQIYALNHVRLDVYAPHLAPRVPDGRAFMVTANLMAPLGR-G 371
pdb|4UDP|     ----------------------VDPATPSDLFLHIGADPVSG--LASAVFWVNKPSST-G 377
pdb|3T37|     -------------------QPEIVVGCGVAPIVSESFPAPAAGSAYSLLFGITHPTSR-G 385
pdb|3LJP|     ---------------RPDLMMHYG-SVPFDMNTLRHGYPTTEN-GFSLTPNVTHARSR-G 386
pdb|4YNT|     VKVSN--GHMKQEDLERLYQLQFDLIVKDKVPIAEILFHPGGGNAVSSEFWGLLPFAR-G 423
pdb|1CF3|     VARG---GFHNTTALLIQYENYRDWIVNHNVAYSELFLDTAG--VASFDVWDLLPFTR-G 434
pdb|3Q9T|     RKAKAANGGKDPFSPLGQPHFELDFVCMFGTAFQWHFPTPKTGDHLTVVVDLVRPISDPG 431
pdb|3FIM|     RLPSNSSIFQTFPDPAAGPNSAHWETIFSNQWFHPAIPRPDTGSFMSVTNALISPVAR-G 423
pdb|6H3O|     KEMGPEFNELWNRYFKDKPDKPVMFGSIVAGAYADHTLLPPGK-YITMFQYLEYPASR-G 432
pdb|1JU2|     ------------------------FTTPPFGFFPSSSYPLPNSTFAHFASKVAGPLSY-G 369

                     490       500       510       520       530       540
              ....|....|....|....|....|....|....|....|....|....|....|....|
Query SapB    TVTAVSPDP-AVPPRIAIDYTAHEE--DARRLRAGVALCWELLGQAPFTAL--------- 419
pdb|4UDP|     WLKLKDADP-FSYPDVDFNLLSDPR--DLGRLKAGLRLITHYFAAPSLAKYGLALALS-- 432
pdb|3T37|     SVRISGPEL-GDRLIIDPAYLQTGR--DRERFRRALEASRTIGHRDELAGWR-------- 434
pdb|3LJP|     TVRLRSRDF-RDKPMVDPRYFTDPEGHDMRVMVAGIRKAREIAAQPAMAEW--------- 436
pdb|4YNT|     NIHISSNDP-TAPAAINPNYFMFEW--DGKSQAGIAKYIRKILRSAPLNKL--------- 471
pdb|1CF3|     YVHILDKDPYLHHFAYDPQYFLNEL--DLLGQAAATQLARNISNSGAMQTY--------- 483
pdb|3Q9T|     EVTLNSADP-FQQPNINLNFFANDL--DIIAMREGIRFSYDLLFKGEGFKD--------- 479
pdb|3FIM|     DIKLATSNP-FDKPLINPQYLSTEF--DIFTMIQAVKSNLRFLSGQAWADF--------- 471
pdb|6H3O|     KIHIKSQNP-YVEPFFDSGFMNNKA--DFAPIRWSYKKTREVARRMDAFRGELTSHHPRF 489
pdb|1JU2|     SLTLKSSSNVRVSPNVKFNYYSNLT--DLSHCVSGMKKIGELLSTDALKPYKVEDLPG-- 425

                     550       560       570       580       590       600
              ....|....|....|....|....|....|....|....|....|....|....|....|
Query SapB    --------------TKEIIDVDG---------------------------RTVESPARLD 438
pdb|4UDP|     -------------RFAAPQPGGPLLN------------------------DLLQDEAALE 455
pdb|3T37|     ---------------ERELLPG-----------------------------TPNSAAEMD 450
```

```
pdb|3LJP|     --------------TGRELSPG----------------------------VEAQTDEELQ 454
pdb|4YNT|     --------------IAKETKPGLSEI------------------------PATAADEKWV 493
pdb|1CF3|     --------------FAGETIPGD-NL------------------------AYDADLSAWT 504
pdb|3Q9T|     -----------------LVESEYPWE------------------------MPLDSDKEMH 498
pdb|3FIM|     -----------------VIRPFDPRL------------------------RDPTDDAAIE 490
pdb|6H3O|     HPASPAACKDIDIETAKQIYPDGLTVGIHMGSWHQPSEPYKHDKVIEDIPYTEEDDKAID 549
pdb|1JU2|     --------------VEGFNILGIPLP------------------------KDQTDDAAFE 447

                       610       620       630       640       650       660
              ....|....|....|....|....|....|....|....|....|....|....|....|
Query SapB    AYVREAARTAHHPMGTARMGRPGDPGAVVDDRLRVHGVQGLRVADASVVPVPVRVNTNLL 498
pdb|4UDP|     RYLRTNVGGVWHASGTARIGRADDSQAVVDKAGRVYGVTGLRVADASIMPTVPTANTNLP 515
pdb|3T37|     DFIARSVITHHHPCGTCRMGK--DPDAVVDANLRLKALDNLFVVDASIMPNLTAGPIHAA 508
pdb|3LJP|     DYIRKTHNTAYHPVGTVRMGAVEDEMSPLDPELRVKGVTGLRVADASVMPEHVTVNPNIT 514
pdb|4YNT|     EWLKANYRSNFHPVGTAAMMP-RSIGGVVDNRLRVYGTSNVRVVDASVLPFQVCGHLVST 552
pdb|1CF3|     EYIPYHFRPNYHGVGTCSMMP-KEMGGVVDNAARVYGVQGLRVIDGSIPPTQMSSHVMTV 563
pdb|3Q9T|     RAVLDRCQTAFHPTGTARLSK-NIDQGVVDPKLKVHGIKKLRVADASVIPIIPDCRIQNS 557
pdb|3FIM|     SYIRDNANTIFHPVGTASMSPRGASWGVVDPDLKVKGVDGLRIVDGSILPFAPNAHTQGP 550
pdb|6H3O|     DWVADHVETTWHSLGTCAMKP-REQGGVVDKRLNVYGTQNLKCVDLSICPDNLGTNTYSS 608
pdb|1JU2|     TFCRESVASYWHYHGGCLVGK------VLDGDFRVTGINALRVVDGSTFPYTPASHPQGF 501

                       670       680       690       700
              ....|....|....|....|....|....|....|....|...
Query SapB    ALALGERAAAW-------------------------------- 509
pdb|4UDP|     TLMLAEKIADAILTQA--------------------------- 531
pdb|3T37|     VLAIAETFARQYHHHHHH------------------------- 526
pdb|3LJP|     VMMIGERCADLIRSARAGETTTADAELSAALA----------- 546
pdb|4YNT|     LYAVAERASDLIKEDAKSA------------------------ 571
pdb|1CF3|     FYAMALKISDAILEDYASMQ----------------------- 583
pdb|3Q9T|     VYAVGEKCADMIKAEHKDLY----------------------- 577
pdb|3FIM|     IYLVGKQGADLIKADQ--------------------------- 566
pdb|6H3O|     ALLVGEKGADLIAEELGLKIKTPHAPVPHAPVPTGRPATQQVR 651
pdb|1JU2|     YLMLGRYVGIKILQERSASDLKILDSLKSAASLVL-------- 536
```

Figure S21. Sequence alignment of proteins related to SapB. Possible catalytic histidine is marked in with a red square.

Supplementary Tables

Table S1: X-ray data and refinement statistics

Dataset	**SapH**
PDB code	7QZJ
Beamline	BioMAX, MAX IV laboratory
Space group	$P2_12_12_1$
Unit cell	
a,b,c (Å)	62.6 63.2 211.4
Resolution (Å)[a]	44.47-1.55 (1.58-1.55)
No. of unique reflections	121033 (5292)
I/σ(I)	11.1 (1.5)
Redundancy	6.4 (2.9)
Completeness (%)	98.8 (88.7)
R_{merge}	0.079 (0.598)
R_{pim}	0.046 (0.509)
CC(1/2)	0.997 (0.679)
Wilson B- value (Å^2)	15.8
Refinement	
R	0.160
R_{free}	0.182
Number of atoms / B-factor (Å^2)	
Overall	7267 / 24.4
Protein	6326 / 22.7
PLP (cofactor)	32 / 20.9
Glycerol	12 / 50.6
Water	894 / 35.9
r.m.s.d. from ideal geometry	
Bond length (Å)	0.009
Bond angles (deg.)	1.42
Ramachandran Plot (%)	
Residues in preferred regions	816 (96.3 %)
Residues in allowed regions	30 (3.5 %)
Outliers	1 (0.1 %)

a: Values in parenthesis are for the highest resolution shell.

Table S2. Primer sequences used to introduce mutations in SapH.

SapH_K289H_forward:	GTTACCATGAGC**CAT**GGTATTAGCG
SapH_K289H_reverse:	CAGATCCGGTGTAACACCATCA
SapH_W32A_forward:	GTGGCATCCG**GCG**TCAAGCGTTC
SapH_W32A_reverse:	AGATGACGACGATCACGTTCACGT
